# The posterior crus II cerebellum is specialized for social mentalizing and emotional self-experiences: a meta-analysis

**DOI:** 10.1093/scan/nsaa124

**Published:** 2020-09-05

**Authors:** Frank Van Overwalle, Qianying Ma, Elien Heleven

**Affiliations:** Department of Psychology and Center for Neuroscience, Vrije Universiteit Brussel, Belgium; Department of Psychology and Center for Neuroscience, Vrije Universiteit Brussel, Belgium; Department of Psychology and Center for Neuroscience, Vrije Universiteit Brussel, Belgium

**Keywords:** mentalizing, emotions, cerebellum, functional neuroimaging, review, meta-analysis

## Abstract

This meta-analysis explores the role of the posterior cerebellum Crus I/II in social mentalizing. We identified over 200 functional magnetic resonance imaging (fMRI) studies via NeuroSynth that met our inclusion criteria and fell within bilateral Crus II areas related to ‘sequencing’ during mentalizing (coordinates ±24 −76 −40; from earlier studies) and mere social ‘mentalizing’ or self-related emotional cognition (coordinates ±26 −84 −34; from NeuroSynth), located in the cerebellar mentalizing network. A large majority of these studies (74%) involved mentalizing or self-related emotional cognition. Other functions formed small minorities. This high incidence in Crus II compares very favorably against the lower base rate for mentalizing and self-related emotions (around 35%) across the whole brain as revealed in NeuroSynth. In contrast, there was much less support for a similar role of Crus I (coordinates −40 −70 −40 from earlier ‘sequencing’ studies) as only 35% of the studies were related to mentalizing or self-related emotions. The present findings show that a domain-specific social mentalizing functionality is supported in the cerebellar Crus II. This has important implications for theories of the social cerebellum focusing on sequencing of social actions, and for cerebellar neurostimulation treatments.

## Introduction

For a long time, it was believed that the cerebellum is exclusively involved in motor control. During the last decades, however, a paradigmatic shift took place supported by empirical evidence, indicating that the cerebellum also supports non-motor mental functions (Schmahmann *et al.*, 2019), especially in the posterior lobe, which is evolutionary younger (Lent *et al.*, 2012). Even more recently, accumulating evidence suggests that the posterior cerebellum also supports social cognition (Van Overwalle *et al.*, 2014, 2015a). Social cognition is the process of perceiving and interpreting the behavior and state of mind of people, including the self (Amodio and Frith, 2006; Van Overwalle, 2009; Van Overwalle and Baetens, 2009). One of the most advanced human social cognitive functions involves interpreting a person’s mind, termed ‘mentalizing’. It requires insight in the mental state of another person or the self, ranging from understanding concrete here-and-now intentions, causes, emotions and beliefs, to abstract social inferences in terms of personality traits (Spunt *et al.*, 2010; Meyer *et al.*, 2012; Spunt and Lieberman, 2012; Baetens *et al.*, 2013), past or future autobiographic events (Svoboda *et al.*, 2006; Spreng *et al.*, 2008; Martinelli *et al.*, 2012), as well as hypothetical thoughts (Van Hoeck *et al.*, 2013). Distortions in mentalizing are considered to cause anomalies in social and affective functioning, such as in autism spectrum disorder, schizophrenia, paranoia and pre-frontal syndromes (D’Mello *et al.*, 2015; Mothersill *et al.*, 2016; Stoodley, 2016).

Although neuroimaging studies on mentalizing focused mainly on the cerebral cortex, more specifically the mentalizing/default network (Van Overwalle, 2009; Van Overwalle and Baetens, 2009; Yeo *et al.*, 2011; Schurz *et al.*, 2014), a seminal meta-analysis by Van Overwalle and colleagues (Van Overwalle *et al.*, 2014, 2015a) provided novel evidence that mentalizing processes are also sub-served by the posterior cerebellum, which is part of the mentalizing/default network of the cerebellum (Buckner *et al.*, 2011). Mentalizing should be distinguished from mirroring, which refers to social understanding by directly observing human bodily motion (of limbs, arms, hands, etc.). In mirroring, social understanding is limited to inferring the goal of human biological movement by direct matching to a representation in one’s memory of own actions and their goals (Gallese *et al.*, 2004; Keysers and Perrett, 2004; Keysers and Gazzola, 2007; Uddin *et al.*, 2007). Mirroring is supported by the mirror network, a part of the sensorimotor network located in the cerebrum (Yeo *et al.*, 2011) and anterior cerebellum (Buckner *et al.*, 2011), which falls beyond the scope of the present meta-analysis.

### Mentalizing in the posterior cerebellum

Although consensus is growing that mentalizing is sub-served by the posterior cerebellum (Van Overwalle *et al.*, 2014, 2015a, 2015b), it is unclear whether this process is unique and specialized in some areas in the posterior cerebellum. Are some areas in the posterior cerebellum uniquely activated during this mentalizing process, or are many other processes sub-served? If specific areas in the posterior cerebellum are preferentially engaged in mentalizing, which mentalizing functions are mostly supported? The aim of this paper is to address these questions by conducting a meta-analysis of cerebellar areas that are involved in social mentalizing and other functions. For this, we use a somewhat unusual strategy: Instead of screening all social studies and looking up which cerebellar areas are activated, we turn this classic procedure upside down and select specific areas known to be strongly involved in social cognition, and look at all the studies, irrespective of domain, that reported activation in these areas. This is novel, because previous meta-analyses demonstrated only that the cerebellum played a role in social cognition, but did not focus on the relative contribution of the posterior cerebellum during social cognition and mentalizing in particular, or on specific mentalizing functions that it may underlie.

There is indeed evidence revealing that the posterior cerebellum might not be preferentially engaged for social cognition after all. An early functional meta-analysis of the cerebellum (Stoodley and Schmahmann, 2009; E *et al.*, 2014) did not report social functions, but pointed to a plethora of other non-social processes that the cerebellum might support. These involved, apart from the classic function of motor perception and execution, other cognitive functions such as semantics, language and executive control. The meta-analysis by Van Overwalle and his team (Van Overwalle *et al.*, 2014, 2015a) provided evidence that mentalizing is sub-served by the posterior cerebellum, but did not investigate how unique this process is. A recent meta-analysis (Guell *et al.*, 2018) provided evidence for social cognition in the cerebellum, but the task was limited to the perception of biological motion of geometric shapes, which is not very representative of human social mentalizing. Recent studies with cerebellar patients documented dysfunctions in lower and higher levels of mentalizing, but did not relate each dysfunction with specific cerebellar areas (Clausi *et al.*, 2019; Van Overwalle *et al.*, 2019a). In sum, although earlier work demonstrated that the cerebellum is involved in social cognition, it did not address the question of how unique this involvement is in comparison with other psychological processes, nor did it compare systematically which distinct mentalizing processes are supported by the cerebellum.

### Social action sequences in the posterior cerebellum

To answer these questions, it is instructive to briefly review recent developments on the theoretical role that the cerebellum might play during mentalizing. There is general agreement that the main function of the cerebellum involves motor implementation, monitoring and automatization, through the construction of implicit internal models of motor behavior and its anticipated somatosensory consequences. It has been suggested that during evolution, a more advanced function developed whereby the cerebellum constructs internal models of pure mental processes in the form of event sequences, without involvement of overt movements and somatosensory responses (Ito and Schuman, 2008; Leggio *et al.*, 2011; Pisotta and Molinari, 2014). This learning and automatization of action sequences in internal cerebellar models allow to send out immediate error signals when unexpected events or environmental changes occur. Applied to social cognition, this evolutionary recent function may support intuitive social understanding of human action and facilitate corrective insight and adaptation to novel social circumstances (Heleven *et al.*, 2019; Van Overwalle *et al.*, 2019b). Internal cerebellar models may also allow humans to plan actions in advance and to engage in social interaction, by anticipating various potential sequences of responses of other agents and alternative ways to reach one’s goal. In support of this view, several functional magnetic resonance imaging (fMRI) connectivity studies confirmed that there are strong links between the posterior cerebellum and key mentalizing areas in the cerebral cortex, such as the temporo-parietal junction (TPJ) and the medial frontal cortex (mPFC) (Van Overwalle *et al.*, 2015b, 2017, 2019c, 2020; Heleven *et al.*, 2019; Van Overwalle and Mariën, 2016).

If these theoretical claims are correct, then mentalizing might engage the cerebellum most strongly when predicting an appropriate sequence of social actions that require the understanding of an agent’s mental state. A key aspect of mentalizing is that observers should be able to understand another person’s belief even when that belief contradicts reality. This aspect is investigated during false belief tasks: unbeknownst by a protagonist, an object is displaced and the critical question is where the protagonist will look for the object (e.g. upon his or her return). To illustrate, if Anne removes Sally’s candy from a basket to a box during Sally’s absence, where will Sally look for her candy when she returns? It takes children up to about 4 years before they can adequately respond to this task by pointing to the original location of the object (where the protagonist Sally saw it last: the basket) rather than where the object is currently located (the box). The original location (involving the correct response) is termed ‘false’ because it refers to a situation that does not reflect the current ‘true’ location.

In a recent pilot study (Van Overwalle *et al.*, 2019a), patients with generalized cerebellar lesions showed the greatest impairments compared to healthy control participants when they had to generate the correct order of four cartoon-like pictures that required the understanding of false beliefs. There were no significant impairments for generating the correct order of routine events that involved mechanical sequences (e.g. a car hitting a rock, then hitting and breaking a tree downhill) or social scripts (e.g. going to the groceries by entering the building, picking items, paying, saying bye and leaving). This seems to indicate that a key social function of the cerebellum involves the correct sequencing of social actions that require the understanding of mental states. A follow-up fMRI study involving healthy subjects (Heleven *et al.*, 2019) also employed this picture sequencing task, and extended it with true beliefs stories (which only require an understanding of another person’s beliefs that coincide with reality) and with a similar verbal version of the task. The results showed that both false and true belief stories in both, pictorial and verbal task versions, engaged the bilateral posterior cerebellum more than non-social mechanical stories. The cerebellar areas recruited during this study are the starting point of our meta-analysis, as discussed below.

### Regions of interest and hypotheses

To test the mentalizing role of the posterior cerebellum, we queried the NeuroSynth database (neurosynth.org) for all studies that fell within pre-designated regions of interest (ROIs), and categorized all studies for their mentalizing and non-mentalizing functionality.

To isolate ROIs specialized for mentalizing in the posterior cerebellum, we started from recent fMRI studies that investigated a key aspect of cerebellar mentalizing: generating the correct sequence of social events that require the understanding of a person’s beliefs. In the study by Heleven *et al.* (2019) previously mentioned, a contrast involving sequencing of true and false belief stories against non-social mechanical stories (including pictorial and verbal stories; total n = 73) revealed activation in the right posterior cerebellum Crus II with Montreal Neurological Institute (MNI) coordinates 25 −75 −40 (see also Van Overwalle *et al.*, 2020). Exactly the same right Crus II ROI was also identified in an earlier connectivity study by Van Overwalle and Mariën (2016; see also Van Overwalle *et al.*, 2019c) that pooled 5 studies (total n = 91) investigating the role of the cerebellum in abstract social reasoning, including person trait inferences and hypothetical counterfactuals.

**Fig. 2.**
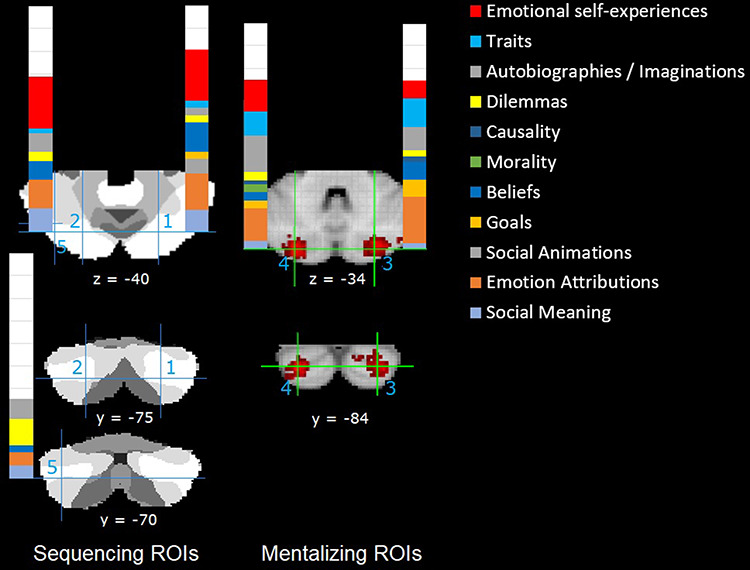
Regions of interest and results of the meta-analysis for the ‘Mentalizing’ and ‘Emotional self-experiences’ Task (sub)categories (the remaining task categories are indicated by white bars). ROIs taken from a top view (z = −40; −34) and a back view (y = −75; −70; −84). The sequencing ROIs 1, 2 and 5 are superimposed on the parcellation of 7 networks from Bruckner et al. (2011), where the white area reflects the mentalizing/default network, and the neighboring light grey area the executive network. The mentalizing ROIs 3 and 4 are taken from NeuroSynth. ROIs 1–4 are in Crus II, ROI 1 with MNI coordinates 25 −75 −40 (Van Overwalle and Mariën, 2016; Van Overwalle *et al.*, 2019c 2020), ROI 2 as it is the left counterpart with MNI coordinates −25 −75 −40, ROIs 3 and 4 with MNI coordinates ±26 −84 −34 from the mentalizing (topic 8 and 28) meta-analysis in NeuroSynth. ROI 5 is in the right Crus I with MNI coordinates −40 −70 −40 (Van Overwalle *et al.*, 2019c).

Given this evidence, we defined this right Crus II peak as our primary ROI 1 together with its left mirror location (ROI 2), and denoted these as ‘sequencing’ ROIs given their potential role during social action sequencing (see Figure 2). Note that these two ‘sequencing’ ROIs are clearly located within the mentalizing network demarcated by Buckner *et al.* (2011). They are also quite close to two lateral Crus II cluster peaks reported in a recent meta-analysis by Guell *et al.* (2018; −24 −79 −36; 20 −78 −34; about 6–8 mm away).

In addition, we derived two bilateral Crus II ROIs from the automated social ‘mentalizing’ meta-analyses in NeuroSynth (ROIs 3 and 4 with MNI coordinates ±26 −84 −34, extracted from the 50 topics set in the NeuroSynth database as of July 2018 (topic #8 and #28, which contains 1054 and 951 studies respectively; https://neurosynth.org/analyses/topics/v5-topics-50/8; for more details, see Poldrack *et al.*, 2012). Other extractions that include ‘mentalizing’ as keyword were also available, such as from the 100 topics set (topic #071, which contains 688 studies) and the 200 topics set (topic #145, which contains 478 studies). Although these sets are arguably more precise, they nevertheless contain fewer studies that might be relevant (e.g. topic #071 from the 100 topics set included the top-loading term ‘non-verbal’ and excluded some studies using exclusively verbal material). Moreover, the selected coordinates for ROIs 3 and 4 fall right in the clusters of these alternative topics sets. It is interesting to note that the 200 topics set has two topics that include ‘mentalizing’ as a keyword, one with mentalizing as main topic (#145) identified before and which shows robust cerebellar activity, while the other (#154) involves predominantly social interaction and shows essentially no cerebellar activity.

Because we will use the NeuroSynth database also for identifying studies of interest for our meta-analysis, it might seem that taking these ‘mentalizing’ ROIs 3 and 4 from the same database results in a sort of double dipping. Nonetheless, an independent analysis of their underlying functions is important because it is still unclear which mentalizing and non-mentalizing functions might be related to these two ‘mentalizing’ ROIs 3 and 4, and to what extent these are similar to the other ‘sequencing’ ROIs 1 and 2. Moreover, as we will see later, many studies reporting coordinates in ROIs 3 and 4 do not involve task activations, but indirect neural measures such as connectivity, volume and so on. These are only remotely related to mentalizing processes, and therefore were eliminated from our analysis. Consequently, the present analysis of ROIs 3 and 4 might be considered as an additional validation and deeper scrutiny of the automated NeuroSynth meta-analysis; it will be interesting to learn whether the automated NeuroSynth topical meta-analyses for mentalizing make sense or not.

We hypothesize that these four ROIs are most likely specialized for mentalizing (including emotional self-experience as they refer to mentalizing about the self). If this prediction is correct, we expect a great majority of mentalizing functions to be recruited by these posterior cerebellar areas, in comparison with non-mentalizing functions and in comparison with the base rate of mentalizing functions across the whole brain in the NeuroSynth database. Given the evolutionary role of the cerebellum in motion, we further predict that within the mentalizing functions, the following input material will recruit our ROIs most prominently: observed or verbally described human movements or actions (e.g. facial and bodily expressions; visual information on human actions), which induce mental inferences (e.g. on other’s emotional states and true or false beliefs), and perhaps also remembered or imagined human actions (e.g. autobiographic memories), based on the assumption that even (static) pictures and verbal descriptions may easily generate notions of dynamic action. In contrast, other input such as the mere physical presence of humans or judgments without any sequence of events will recruit our ROIs less likely, such as trait judgments based on trait adjectives (i.e. they imply past actions but do not directly refer to a sequence of actions).

Finally, from the study mentioned earlier by Heleven *et al.* (2019) on sequencing true and false belief stories, another peak was identified using the same procedure involving a contrast of true and false belief stories against non-social mechanical stories, in the left posterior cerebellum Crus I located somewhat more peripherally with MNI coordinates −40 −70 −40 (see also Van Overwalle *et al.*, 2020). This peak served as ROI 5. To the extent that ROI 5 is very close to the executive network (Buckner *et al.*, 2011) as can be observed in Figure 2, it might reveal relatively less empirical support for mentalizing functionality, and as such may serve as a sort of baseline to estimate the relative contribution of mentalizing reasoning in the posterior cerebellum in more general.

## Method

### Selection of studies

**Fig. 1.**
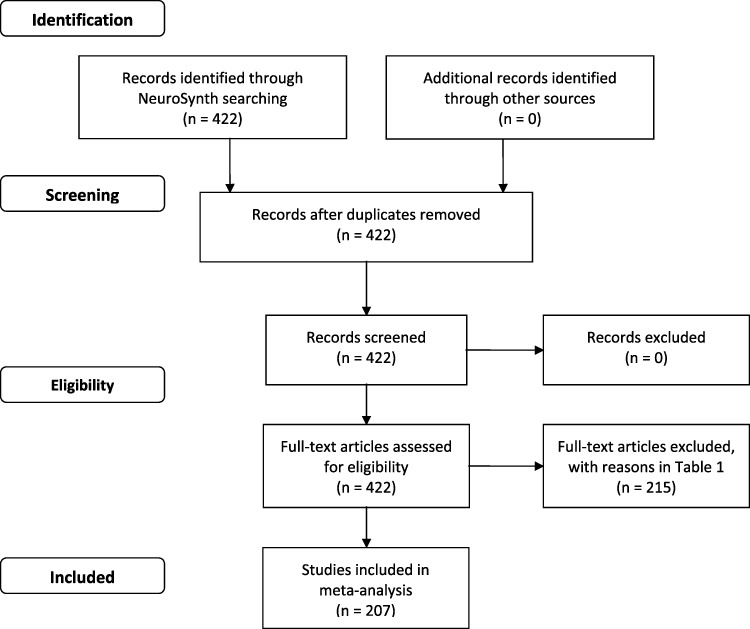
Prisma diagram of the meta-analysis on Mentalizing and Emotional self-experiences.

The fMRI studies reviewed in the current meta-analysis were taken from the NeuroSynth database (neurosynth.org). A study was defined as a single fMRI experiment, and all coordinates from all task-related analyses in each study were eligible (in the same manner as in NeuroSynth). No attempt was made to include studies from other databases, because as far as we are aware, NeuroSynth is currently the only one that allows to select studies on the basis of pre-defined ROIs. An overview of the identification of studies, their screening and eligibility is given in Figure 1.

As detailed in the introduction, we selected five ROIs in the posterior cerebellum that were related to mentalizing (Figure 2). Note that for the right ‘sequencing’ ROI 1, the data from Heleven *et al.* (2019) were pooled across pictorial and verbal sequencing tasks. A contrast comparing sequencing of true and false belief stories against non-social mechanical stories (*P* < 0.05 FWE-corrected) resulted in a cluster peak with MNI coordinates 25 −75 −40 after rounding to the next 5 mm, and which matched exactly with the ROI of Van Overwalle and Mariën (2016). Afterward, in NeuroSynth, the MNI coordinates of the right and left ‘sequencing’ ROIs were automatically rounded to even values ±24 −76 −40.

We identified all studies in NeuroSynth within a radius of 6 mm around the coordinates of all five ROIs. From these, we selected studies that fulfilled the following inclusion criteria:

- MRI studies that reported functional results. Consequently, MRI studies involving only resting state or connectivity analyses, structural or volumetric measurements, an independent or principal component analysis, or coupled with EEG measurements, were excluded. PET studies were also excluded.
- fMRI results expressed in MNI template (Collins *et al.*, 1994) to make sure that the reported peaks fell within the 6 mm radius. Studies with Talairach coordinates (Talairach and Tournoux, 1988) were excluded because, contrary to the claim on the NeuroSynth website, more often than not, Talairach coordinates were not converted to MNI coordinates. In addition, converting coordinates from Talairach to MNI might be potentially very biased for the cerebellum, because this area is the furthest away from the zero brain coordinates. Moreover, fMRI studies with incorrectly reported coordinates in NeuroSynth or in the original study (i.e. not located within the anatomical area indicated) were also excluded, since they did not fell within the 6 mm radius.
- fMRI results that involved unmedicated healthy participants, either adults or adolescents (i.e. > 10 years old, see also Van Overwalle *et al.*, 2015b). Clinical studies were included if they reported the separate and independent results of healthy control participants. In contrast, studies with children (<10 years old) or fMRI coordinates resulting from statistical interactions with patients, medication, a chemical substance or genetic measurements were excluded.
- fMRI studies that involved a comparison against an adequate control condition or a parametric regression analysis. This excluded, for instance, comparisons between left and right hemispheres (Stevens *et al.*, 2005) and fMRI coordinates that showed more activation during a control rest condition than an experimental condition (e.g. Schlerf *et al.*, 2012; Spreng *et al.*, 2014), except when predicted (e.g. Ishizu, 2014).
- fMRI coordinates from empirical findings for the whole (sub)sample. Consequently, coordinates from individual participants were excluded, as well as from meta-analyses and review studies.

Of the more than 400 studies initially identified, these criteria led to the inclusion of 207 (49%) valid studies in total. Table 1 lists the criteria for exclusion and the number of studies involved (see also Supplementary Table S1), as well as some study/demographic variables of the included studies (i.e. gender, age and sample size without participants excluded from the analysis). A chi-square test revealed that none of the ROIs showed an unequal distribution of the exclusion criteria (relative to the total number of studies excluded in each ROI), *P* > 0.99.

**Table 1.** Criteria and number of excluded studies; demographics of included studies

		Regions of interest (radius 6 mm)												
		Sequencing			Mentalizing					Sequencing				
		ROI 1	ROI 2		ROI 3	ROI 4				ROI 5				
		Right Crus II	Left Crus II		Right Crus II	Left Crus II				Left Crus I				
		24 −76 −40	−24 −76 −40		26 −84 −34	−26 −84 −34				−40 −70 −40			Studies	%
Excluded studies														
Criteria for exclusion:														
Technique and analysis														
	Volume	3	1		4	3				3			14	3%
	Connectivity	7	2		10	8				4			31	7%
	Resting state	2	4		9	2				7			24	6%
	Meta-analysis	0	0		1	1				2			4	1%
	Other (SPECT, EEG, etc.)	0	1		1	3				3			8	2%
Population and non-psychological factors														
	Patients	7	13		9	10				12			61	14%
	Medication/substance	0	0		1	1				1			3	1%
	Genetic interaction	1	0		1	0				0			2	0%
Coordinates														
	Incorrect coordinates	1	1		2	0				1			5	1%
	Talairach coordinates	11	8		19	8				9			55	13%
Other														
		1	2		3	1				1			8	2%
Number of excluded studies		33	32		60	37				43			215	51%
% of excluded studies		40%	52%		52%	49%				56%				
Included studies														
Demographics (means):														
	Number of participants	23.0	45.6		40.6	45.3			20.2					
	Males	12.2	20.1		18.1	22.1			11.8					
	Mean age	28.2	27.3		26.6	25.7			25.2					
Number of included studies		48	30		56	39			34				207	49%

A chi-square test revealed that none of the ROIs showed an unequal distribution of the exclusion criteria, *P* > 0.08. SPECT = Single Photon Emission Computed Tomography; EEG = Electro-encefalography

Note that some studies were identified for more than one ROI. This is due to the fact that the centers of the ROIs are only 11 mm apart and the 6 mm radius in each ROI thus causes some overlap. To illustrate, several studies appear in multiple ROIs with different coordinates (e.g. Bzdok *et al.*, 2012, appears in ROI 2, ROI 4 and ROI 5; Korn *et al.*, 2012, appears in ROI 3 and ROI 4; 2014, appears in ROI 3 and ROI 4; Schnell *et al.*, 2011, appears in ROI 3 and ROI 4; Wilson *et al.*, 2008, appears in ROI 1 and ROI 2) or with the same set of coordinates (e.g. Hartwright *et al.*, 2015 appears in ROI 1 and ROI 3).

### Classification of studies

Next, we identified several task categories that were most similar among a set of studies. The classification of each study into a task category was determined by the description of task, stimuli of the main condition (in the contrast or regression), instructions and contrast between experimental and control conditions, using similar criteria as in earlier meta-analyses by Van Overwalle and colleagues (Van Overwalle, 2009; Van Overwalle and Baetens, 2009; Van Overwalle *et al.*, 2014) and Schurz *et al.* (2014; see Table 2 for some examples, Supplementary Table S1 for more details).

The criteria for mentalizing, as well as for non-mentalizing functions, were detailed as follows:

**Table 2.** Examples of studies for all major task and mentalizing categories

Authors	Year	Stimulus modality	General category	Mentalizing content	Stimuli of main condition	Task or instructions	Contrast (>) or regression	
Groen	2010	Auditory	Mentalizing	Social Meaning	Human actions (H_method_ > 80%)	Detecting sentences with pseudowords	Normal sentence > Speech-like noise	
Johnston	2013	Visual (Pictures/Video)	Mentalizing	Emotion Attributions	Facial emotional expressions	Making emotion judgment (Fear/Surprised) or gender judgment (Female/Male).	Categorizing emotion > Gender	
Jack	2015	Visual (Animations)	Mentalizing	Social Animations	Shape motion (goal-directed and social)	Passively watching shapes moving in goal-directed or social ways (e.g. chasing other, fighting, dancing together, hiding).	Goal-directed > Random motion	
Spunt	2011	Visual (Videos)	Mentalizing	Goals	Human actions	Answering questions about: ‘what/why/how is the agent doing’	Why > What > How questions	
Lewis (Exp. 2)	2017	Verbal	Mentalizing	Beliefs	Human actions	Answering questions about agents who have different beliefs about other agents	Belief > Facts	
Schneider	2013	Visual	Mentalizing	Morality	Human actions (moral dilemma)	Judging a solution related to individual gain *vs* collective losses	Individual *vs* collective moral dilemma > control	
Fukushima	2013	Visual and Auditory	Mentalizing	Causality	Square changes in positions and color	Judging self-agency, whether changes in a target are caused by own actions	Movements caused by self > not caused by self	
Servaas	2015	Visual (Videos)	Mentalizing	Dilemmas	Self-decision	Accepting or rejecting monetary proposals by opponent.	Unfair > Fair rejection (for unfair proposals)	
Nawa	2014	Verbal	Mentalizing	Autobiographies	Autobiographic memories	Retrieving memories of negative or positive events in personal lives	Autobiographic memory > Counting control	
Matsunaga	2017	Verbal and Visual (Pictures)	Mentalizing	Imaginations	Human actions	Imaging life events experienced alone or with a friend, and rating happiness	With friend > Alone	
Debbane	2017	Verbal	Mentalizing	Traits	Trait adjectives	Attributing positive or negative traits to self or best (same-sex) friend	Trait judgments > Counting syllables control	
Engen	2015	Visual (Videos)	Emotional	—	Human actions	Rating positive and negative emotions about distressing and neutral video clips while implementing a Reappraisal or Compassion regulation technique.	Emotion regulation: Reappraisal > Compassion	
Seghier	2011	Verbal/Visual	Semantic	—	Words/Pictures (H_example_ = 0%)	Indicating which item (word/picture) is most semantically related to target item	Semantic > Perceptual matching	
de Diego Balaguer	2006	Verbal	Linguistic	—	Regular and Irregular verbs	Producing the present tense form of the verb or repeating the infinitive form	Nonce verb inflection > Irregular verb inflection	
Fogel	2014	Visual	Motor	—	Finger tapping	Performing a motor sequence by tapping the fingers	Motor sequence training	
Beudel	2009	Visual (Animations)	Motor perception	—	Ball movements	Judging the speed of a ball moving or the place where ball disappears	Speed > Place at stop	
Evans	2002	Somatosensory	Somatosensory	—	Urge to breath (Air hunger)	Rating urge to breath	Urge to breath	
Mutschler	2007	Auditory	Music	—	Melodies (Piano)	Listening to melodies that were played or listened to earlier multiple times	Number of trials needed to learn melodies	
Liu	2006	Visual	Cognitive	—	Stroop task	Identifying ink color while ignoring the meaning of same/different colored words	One-word (same) > Four-word (different) Stroop task	

H = Percentage of human or humanized animals in the stimulus material based on the information provided (source indicated by subscript).

- **Mentalizing**: First, we excluded (visually) observed limb (e.g. hand, arm, leg) movements because this may induce goal inferences based solely on social ‘mirroring’ processes, which do not require mentalizing. Second, in line with the task categories specified by Van Overwalle (2011) and other meta-analyses listed above, and based on the stimulus material of the main condition and the statistical analysis (i.e. contrast or parametric modulation), we identified mentalizing through a number of content sub-categories. These sub-categories are listed below, loosely ranged from concrete (i.e. here and now) to abstract in the line of Van Overwalle *et al.* (2014). If more than one content sub-category was possible, we indicated in the Supplementary Table S1 the higher level of abstraction:
- **Social Meaning** involves comprehension of human actions and narratives, minimally about the goal-direction of the action, without being asked explicitly about the specific mental state of the agent (e.g. X goes to the movies; Van Overwalle, 2011). This is based on the assumption that understanding ‘rational actions … requires attributing intentions to the protagonist of a story’ (Schurz *et al.*, 2014, p. 19). There is a wealth of behavioral and neuroimaging research showing that mental states are spontaneously inferred on the basis of human behavior (Ma *et al.*, 2011; Uleman *et al.*, 2012; Kovács *et al.*, 2014; Schneider *et al.*, 2017; Bott *et al.*, 2019). Consequently, the particular statistical contrast is of less importance here and need not compare mentalizing *vs* non-mentalizing conditions, as long as meaningful understanding is extracted from the information in the main condition, and less so in the control condition. For example, when participants are passively listening or watching narratives about humans with the instruction that they will be asked questions about the plot, and the statistical contrast compares language comprehension (narratives) *vs* rest (blank screen) (Wilson *et al.*, 2008), or when passively listening to narratives without further instructions and the contrast shows increased activation during semantic anomalies (Tesink *et al.*, 2011).
- **Emotion attributions** to others (including preferences of others), answering questions such as ‘what is X feeling/liking?’ or ‘who is happier/angrier?’, which are often based on stimuli involving facial and body expressions as well as emotional ratings of human actions. There is ample evidence that mentalizing ‘is implicated in consciously reflecting upon or regulating … someone else’s affective states (e.g. X smiles), which involves empathizing with someone’s feelings’ (Van Overwalle, 2011, p. 1595). Note that emotional experiences by the self are categorized in a separate task category (see below).
- **Social Animations** of geometrical shapes (e.g. two triangles) of which ‘the movements portrayed actions which are typical for an intentional or social interaction’ (Schurz *et al.*, 2014, p. 16).
- **Goals** are implied given human actions and human narratives involving a target object or state when answering questions such as ‘why is X doing this, what goal is X pursuing?’
- **Beliefs** of others, answering questions such as ‘what is X thinking/believing?’ often during human actions and human narratives. This often involves ‘false belief stories as the prototypical problem for theory of mind reasoning’ (Schurz *et al.*, 2014, p. 13).
- **Morality** by others, answering questions such as ‘what is X’s responsibility?’ (e.g. X harms Y purposefully), often during recounted or observed human actions and narratives. Describes ‘events of moral (in)justice with as main element the intentional act of wrongdoing or help’ (Van Overwalle, 2011, p. 1595).
- **Causality** by others (i.e. agency), answering questions such as ‘what is X causing/controlling?’ or ‘what caused the behavior of X?’, also during recounted or observed human actions and narratives. For example, when a person has varying levels of control over reaching a target, participants need to be aware of this and make appropriate causal attributions for successful performance (Chaminade *et al.*, 2012).
- **Dilemmas** under ‘the hypothesis … that feedback from a social partner – indicated by her moves in the game – is spontaneously used to infer her intentions, even if participants are not explicitly told to mindread’ (Schurz *et al.*, 2014, p. 15). Also includes self-decisions in dilemmas without human opponents, because this requires self-reflection about one’s hypothetical future state for each of the options (and rewards) taken and foregone.
- **Autobiographies** or memories about actions of the self and others in a distinct past, future and hypothetical **imaginary** situation, answering questions such as ‘what was/will I be doing?’ (Van Overwalle *et al.*, 2014).
- **Traits** of others and self, based on trait adjectives without action descriptions, answering questions such as what type of person someone is (e.g. how much does ‘smart’ apply to X?) involves ‘conceptual knowledge about persons’ (Schurz *et al.*, 2014, p. 13).

We further excluded from social mentalizing the mere presence of humans while their actions are unclear or undetermined. Note that material that was not considered mentalizing by the researcher(s) conducting the study, was screened on the presence of humans in the stimuli (e.g. humans in the scenes, events, etc.) as determined from the description in the method section (denoted by H_method_ in Table 2 and Supplementary Table S1), examples in the method section (denoted by H_examples_), or the Supplementary Material of the study (denoted by H_suppl_). Any material that contained more than 50% humans in the main experimental condition was included as mentalizing if it fulfilled one of the content criteria described above. For example, if sentences were explicitly screened for similar meaning (i.e. synonyms) and more than 50% of the sentences contained humans who engaged in various actions, this was categorized under the sub-category ‘social meaning’ (Hoffman *et al.*, 2015).

- **Emotional self-experiences** given questions such as ‘How are you feeling?’, or the attribution of an emotion to a situation without a clear interactive social context or actions, even without an explicit instructions to do so. We categorized the emotion category as a separate category in line with the majority of the emotion literature, although strictly speaking, emotional self-experiences or self-judgments involve mentalizing appraisal processes with the self as object rather than another person, also referred to as ‘conceptualization’ (Lindquist and Barrett, 2012) and ‘mentalizing’ (Van Overwalle, 2011). Neuroimaging research shows that emotions show a large overlap with mentalizing (Van Overwalle, 2009, 2011), especially when they involve consciously reﬂecting upon the reconstruction of events and (re)appraisals that lead to the emotion, or reflecting about their good or bad outcomes (Cunningham *et al.*, 2004 2007; Lindquist and Barrett, 2012; Lindquist *et al.*, 2012; Buhle *et al.*, 2013). Therefore, it is very likely that self-related emotions trigger mentalizing appraisals and so recruit the posterior cerebellum. This is supported by the finding that topic #26 in the 50 topics set of NeuroSynth, which is of most relevance, shows a ROI in the right posterior cerebellum roughly at MNI coordinates 26 −78 −34, which is between ROIs 1/2 and 3/4.
- **Semantic** understanding of language, that is, grasping the meaning of words, sentences and narratives. To understand a story or sentence, the comprehension of goals, behaviors and mind of agents is often a prerequisite, so that semantics share a lot of commonalities with mentalizing (Mar, 2011). In line with the screening criteria as described above, studies in this category are purely semantic independent from a social context and contain less than 50% trials with humans.
- **Linguistic** functions involving grammar, spelling (of Western and Eastern characters) and pronunciation. These were all categorized as non-mentalizing.
- **Motor Execution** and **Motor Perception** involve evolutionary older key functions of the cerebellum, typically located in the anterior cerebellum. As noted above, when biological movements were executed by humans (or biological human-like movements by abstract shapes), the perception of these actions was categorized under motor perception as it may trigger social mirroring, rather than mentalizing (Van Overwalle *et al.*, 2014).
- **Somatosensory** refers to sensory functions that respond to changes inside or at the surface of the body.
- **Music** that clearly requires sensory input or motor responses that follow a sequence.
- **Cognitive** refers to a variety of processes such as executive functions to plan and direct goal-oriented behavior, and ignoring or resolving inconsistencies. It also includes memory and numerical operations. When out of a social context, these functions were categorized as non-mentalizing.

Classification of all the studies proceeded in two steps. The initial decision on eligibility, checking of coordinates and classification of task categories, modality, stimuli/material, instructions and contrasts (see examples in Table 2) was made after a first reading of each article by Frank Van Overwalle (FVO), and Qianying Ma (QM) (ROI 1, 2 and 5), or QM alone (ROI 3 and 4). These classifications were then thoroughly checked by FVO. This final check generally confirmed the initial coordinates and the sub-division of the major task categories and stimulus modalities, although minor rephrasing and reinterpretation occurred for the mentalizing sub-categories (stimuli), instructions and contrasts. On the basis of a reviewer’s suggestions, the classifications of the Mentalizing and Emotion categories were checked anew by FVO and QM, leading to some re-categorizations. All these classifications are reported in full in the Supplementary Table S1.

## Results

Some exemplary studies are listed in Table 2, listing all major tasks and mentalizing categories included in this meta-analysis. Supplementary Table S1 lists the details of all individual studies, including task categories, mentalizing content, stimulus modality, stimulus material, instructions and contrasts.

A summary overview of the major task categories is given in Table 3 (top panel), showing the percentage of studies that revealed activation in each of the ROIs. As predicted, in Crus II (ROIs 1–4), the mentalizing category involved the majority of the studies with an average of 57% over all ROIs (ranging from 46% to 67%), while in Crus I (ROI 5) this reduced to 35%. Since the emotional self-experiences category may be considered as mentalizing, we also took both categories together. The reason is that emotional self-experiences involve mentalizing appraisal processes with the self rather than another person as object of judgment. These self-directed processes have been referred to as ‘conceptualization’ (Lindquist and Barrett, 2012) and ‘mentalizing’ (Van Overwalle, 2011). This combined mentalizing and emotional categorization resulted in an average Crus II involvement for 74% of the studies, as compared to 35% for Crus I. In addition, in Crus II a sizeable number of studies also involved semantic, motor-related, musical and cognitive tasks (each no more than 8% on average). In contrast, Crus I revealed a variety of non-mentalizing processes, starting with cognitive tasks as most strongly represented (most often numeric or memory tasks; 18%), followed by tasks related to semantics (15%), somatosensory experience (12%), music (9%), motor processes (6%) and language (6%).

**Table 3.** Major task categories and social sub-categories of the regions of interest, and comparative data on topics in the NeuroSynth database

	Regions of interest (radius 6 mm)											
	Sequencing			Mentalizing					Sequencing			
	ROI 1	ROI 2		ROI 3	ROI 4				ROI 5			
	Right Crus II	Left Crus II		Right Crus II	Left Crus II		Crus II		Left Crus I		NeuroSynth	
Category/Sub-category	24 −76 −40	−24 −76 −40		26 −84 −34	−26 −84 −34		Means		−40 −70 −40		Topic #	Studies
	General categories											
Mentalizing	46%	57%		61%	67%		*57%*		35%		#8 #28 (social) #40 (face)	20%
Emotional self-experiences	23%	23%		14%	8%		*17%*		0%		#26 (emotional)	15%
Semantic	15%	0%		13%	0%		*7%*		15%		#38 (semantic)	9%
Linguistic	0%	0%		0%	0%		*0%*		6%		#37 (language)	12%
Motor/motor perception	13%	3%		4%	8%		*7%*		6%		#17 (motor) #45 (motion)	15%
Somatosensory	0%	0%		0%	0%		*0%*		12%		#32 (somatosensory)	6%
Music	2%	0%		2%	10%		*4%*		9%		#6 (auditory)	9%
Cognitive^a^	2%	17%		7%	8%		*8%*		18%		#9 #33 (memory) #18 (number) #11 (learning)	29%
Number of all studies	48	30		56	39		*43.3*		34			14 371
	Mentalizing Content Sub-categories											
Social Meaning	23%	18%		6%	4%		*13%*		17%			
Emotion Attributions	27%	29%		24%	31%		*28%*		17%			
Social Animations	0%	6%		0%	0%		*1%*		0%			
Goals	0%	6%		6%	12%		*6%*		0%			
Beliefs	18%	24%		6%	12%		*15%*		8%			
Morality	0%	0%		6%	0%		*1%*		0%			
Causality	0%	0%		3%	4%		*2%*		0%			
Dilemmas	9%	6%		6%	4%		*6%*		33%			
Autobiographies/imaginations	18%	6%		26%	15%		*16%*		25%			
Traits	5%	6%		18%	19%		*12%*		0%			
Number of mentalizing studies	22	17		34	26		*24.8*		12			
	Motion/Action related Stimuli of Main Condition in Mentalizing											
Facial/body expressions	23%	18%		12%	19%		*18%*		8%			
Human actions	50%	65%		38%	35%		*46%*		42%			
Autobiographic memories	18%	0%		24%	8%		*12%*		8%			
Sum of percentages	91%	82%		74%	62%		*76%*		58%			
Number of mentalizing studies	22	17		34	26		*24.8*		12			

Percentages in the cells refer to the proportion of studies within a category in relation to the total number of all studies (top panel), or total number of mentalizing studies (middle and bottom panels). The NeuroSynth topics were selected from an available set of 50 topics extracted from the abstracts of all articles in the NeuroSynth database as of July 2018. Topic words between parentheses are selected as most representatives from the first two most sampled terms (Poldrack *et al.*, 2012). Italics refer to the means across ROI1 to ROI4 (all Crus II) = mean value of the four Crus II Regions of Interest.

^a^ Includes one ‘Visual’ study in ROI −24 −76 −40.

We explored whether there were any differences between the number of studies in Crus II against Crus I, using a X test assuming an equal distribution across the ROIs. Comparing mentalizing, emotional self-experiences and all other categories combined (Table 3), this difference was highly significant, X (2) = 15.46, *P* < 0.001. We further explored any differences within the four ROIs in Crus II and found an unexpected increase in the right as opposed to the left ROIs for the semantic task category, X (3) = 14.00, *P* < 0.01. These findings are probably related to the contralateral connectivity with left-located languages areas in the cerebrum.

To provide a comparative base rate of the typical number of fMRI studies of each task category, we queried the NeuroSynth database and listed in Table 3 (top panel, far right) the number of available studies under several topics from the set of 50 topics in the database closely related to our task categories (extracted in July 2018; Poldrack *et al.*, 2012). This comparison evidently assumes that the work tabulated in NeuroSynth, and in the field at large, is somehow representative of the distribution of processes in the human brain. Note that when more than one topic was selected, we counted only one time each study contributing to multiple topics (i.e. without duplicates). As can be seen, across the whole brain, all topics ranged between 6% and 29% of all fMRI studies, indicating that the high incidence of mentalizing/emotion studies in Crus II in our study does not result from a higher base rate of this type of studies, but seems to be specific to this area. Although the topics from NeuroSynth are somewhat arbitrarily selected and the reported base rates are therefore suggestive at best, this conclusion is upheld when other relevant topics from NeuroSynth are selected.

Table 3 (middle panel) also provides the mentalizing sub-categories (see also Figure 2), reflecting the content of mentalizing. Consistent with our prediction, for the ROIs in Crus II, the highest percentages are found for mentalizing sub-categories that directly reflect or imply human behavior and movement, including social meaning (13%), explicit goals (6%), beliefs (15%) and autobiographies (16%), with the highest incidence for emotion attribution often via facial/bodily expressions (28%). Higher-order attributions involving morality (1%) and causality (2%) can be considered as higher-order goal attributions referring to human responsibility and causality of goal-driven behavior and may be added to that category. In contrast, as predicted, lower percentages are generally found for mentalizing sub-categories that do not reflect human action sequences, such as trait judgments (12%) and dilemmas (6%). Note, however, that these percentages are not compared against existing base rates, so that they are descriptive at best. Exploratory X tests did not reveal significant differences between the four ROIs of Crus II, *P* > 0.05.

Finally, Table 3 (bottom panel) provides the stimulus categories of the mentalizing studies, which were derived from the stimuli of the main condition in all included studies (see Supplementary Table S1, ‘Stimuli of Main Condition’ columns). Consistent with our prediction that the cerebellum is involved in dynamic sequencing of action and movement, for the ROIs in Crus II, on average the largest proportion of the stimulus material involved human actions (47%) and autobiographic memories of actions (12%), or a total of 59%. If we include facial/bodily expressions (18%), the total proportion raises to a mean 77%. Exploratory X tests revealed significant differences between the Crus II ROIs for autobiographic memories, X (3) = 10.00, *P* < 0.05, showing that these were more activated for the right than left ROIs. Note, however, that the proportion of all action- and emotion-related material was also substantial for the Crus I ROI 5 (58%), suggesting that many mentalizing inferences in both Crus I and II are supported by them.

## Discussion

This meta-analysis explored the functional role of the posterior cerebellum in mentalizing. Given that the ROIs in the bilateral Crus II derived from earlier belief sequencing studies (‘sequencing’ ROIs 1 and 2 with MNI coordinates ±24 −76 −40) and from the NeuroSynth mentalizing meta-analysis (‘mentalizing’ ROIs 3 and 4 with MNI coordinates ±26 −84 −34) are located within the mentalizing network by Buckner *et al.* (2011) and are close to the cluster peaks reported in a recent meta-analysis by Guell *et al.* (2018), we hypothesized that these areas are specialized in mentalizing. Consistent with this prediction, we found that a large majority of 74% of the eligible studies involved mentalizing functions, including emotions attributed to others and self-experienced emotions. Other non-mentalizing functions formed small minorities and were variously related to motor, somatosensory and executive tasks (including music, semantics, memory, numbers, and so on). We found no differences in mentalizing activity between these four ROIs. The high incidence of mentalizing in Crus II is very distinct from the lower base rate across the whole brain as revealed in NeuroSynth.

Consistent with the hypothesis that the cerebellum is involved in action sequence detection, a high incidence of mentalizing sub-categories was based on actual or reconstructed human actions, such as when attributing social meaning (i.e. implicit goals), explicit goals, beliefs and autobiographic memories. Interestingly, the highest incidence was found for emotion attribution, often on the basis of static facial and bodily expressions. Given that emotion attribution and experience has been left out from many recent meta-analyses on mentalizing (e.g. Schurz *et al.*, 2014; Van Overwalle *et al.*, 2014; but not in Guell *et al.*, 2018), more research on the role of the cerebellum in emotion attribution is needed to understand their relationship with mentalizing. This relationship might be complex, as recent studies with cerebellar patients show impairments on social mentalizing, but not necessarily on emotion attribution (Clausi *et al.*, 2019; Van Overwalle *et al.*, 2019a). In contrast, as predicted, lower percentages were generally revealed for mentalizing without explicit human sequencing, such as trait attributions, and dilemmas.

We also hypothesized that the Crus I ROI 5 (with MNI coordinates −40 −70 −40) would reveal less empirical support for mentalizing functionality, because it is located close to the boundary of the mentalizing and executive network (Buckner *et al.*, 2011). Consistent with this expectation, only 35% of the studies recruiting this area were related to mentalizing and self-related emotionality. The other activations were related to a variety of other mental processes, of which the most prominent ones (i.e. cognitive, 18%, and semantic/linguistic, 15%) are consistent with the location of this area close to the executive network. Of critical importance is that the lower degree of mentalizing activations in Crus I, together with the low base rate in the NeuroSynth database, suggests that the high incidence of mentalizing processes in our meta-analysis is related to its specific location in the bilateral Crus II.

The high percentage of mentalizing studies in Crus II is surprising. In an earlier meta-analysis on the cerebellum (Van Overwalle *et al.*, 2014; > 350 studies), activation of the cerebellum was revealed in only about one-third of the social cognitive studies (except for more abstract categories such as traits and autobiographic memories, showing activation in about three-fourth of the studies). However, this lower percentage in past research might be due to several methodological limitations of the studies included. We surmise that many researchers simply neglected (parts of) the cerebellum in the scanning procedure (i.e. window) or scientific reports, as they might have not expected that the cerebellum was important for social functioning. Also surprising is that this earlier meta-analysis revealed most mentalizing in Crus I rather than Crus II, although this might in part be due to shortcomings in the conversion from the original Talairach to MNI coordinates which may have led to underreported Crus II clusters. In contrast, a recent large-scale analysis by Guell *et al.* (G2018; 787 participants) reported about equal recruitment of Crus I and Crus II, but their analysis involved only social animations and no other mentalizing tasks, which seriously limits this finding. Moreover, as noted in the introduction, the high incidence of mentalizing in Crus II is consistent with recent fMRI studies focusing on the connectivity of the cerebellum with the cerebral cortex among healthy adults (Van Overwalle *et al.*, 2019c, 2020) or individuals with autism (Olivito *et al.*, 2018), and the cerebellar network structure proposed by several authors (Buckner *et al.*, 2011; Guell *et al.*, 2018).

The existence of a small incidence of additional functions besides mentalizing in Crus II, such as various sensorimotor and executive functions (including semantics, linguistics and memory) is consistent with similar overlap found between these functions in the meta-analysis of the cerebellum mentioned earlier (Van Overwalle *et al.*, 2014). More work is needed to establish whether these overlapping functions are empirically robust, either at a meta-analytic level or within single studies. Are these overlaps due to specific core operations that support multiple mentalizing and non-mentalizing processes sub-served in these cerebellar areas? Or are mentalizing *vs* non-mentalizing functions separated by loose patchy boundaries in the cerebellum? These are fascinating questions for future research. Perhaps studies that directly compare social information processing (e.g. false beliefs) with non-social controls (e.g. outdated photos), can provide more insight in this question, by revealing areas in the cerebellum common to the underlying reasoning but distinct in their social content. However, from all studies reviewed in this article, none provided sufficient information to answer this question.

### Limitations

The present meta-analysis has a number of limitations. Perhaps the most important limitation is that readers should be aware that we screened only a limited set of ROIs, not the whole Crus I/II area. Thus, it might well be that beyond the ROIs investigated here, some areas in Crus II are less specialized in mentalizing or that some areas in Crus I are more specialized. Moreover, other areas in the cerebellum might be relevant. For instance, lobule VI was revealed by Van Overwalle *et al.* (2014) as another important area involved in self-references and autobiographic memories (often involving the self), and in the large-scale analysis by Guell *et al.* (2018) as involved in social animations. However, this area was also associated with many non-mentalizing tasks in the meta-analysis by E *et al.* (2014) and Guell *et al.* (2018), so that it might be less specialized for social mentalizing.

Second, the selection of studies was based on a single database: NeuroSynth. This was for obvious reasons, namely, to conduct a reverse meta-analysis starting from fMRI studies within a set of pre-determined spheres (ROIs) in the posterior cerebellum.

Third, the studies were limited to MNI coordinates. This was because of the uncertainty surrounding Talairach coordinates, as they were not always converted to MNI coordinates in the NeuroSynth database and thus often fell beyond our pre-determined spheres with 6 mm radius. Because current studies are mostly reported in MNI coordinates, this limitation affected most often older studies.

Fourth, we included emotional self-experiences as a separate task category and not as part of our ‘mentalizing’ studies. Although one might concur that that emotional self-experiences involve somatosensory processes that are available only to the self, and not when processing others, modern views of emotions consider emotional appraisals or conceptualization as mentalizing processes, which are an intimate part of emotions (Lindquist and Barrett, 2012) in which a person turns his or her perspective to the mental state of the self. More generally, many studies categorized as mentalizing did not reflect pure social-cognitive processes and often included elements of emotionality. This is consistent with neuroimaging research showing that emotions show a large overlap with mentalizing (Van Overwalle, 2009, 2011).

Fifth, we used the NeuroSynth database to gauge the base rate of similar task categories across the whole brain. Although the ‘topics’ in NeuroSynth are the closest match to the present task categories, obviously they have been defined and compiled in a completely different manner, so that these comparisons are merely indicative.

Sixth, we found no lateralization in the ROIs considered, except for semantics. This is an issue for further research on the cerebellum, although this might be quite unsuccessful because meta-analyses of cortical areas involved in social mentalizing often failed to find evidence for strong hemispheric differences (e.g. Schurz *et al.*, 2014).

A final limitation concerns the specificity of the mentalizing and self-related emotional processes being studied in this meta-analysis. All socially relevant studies were included, except for two categories—social mirroring and the mere presence of humans (when their actions were unclear). Apart from these two, it may appear that all aspects of social or self-referential cognition fall under the heading of mentalizing. However, this may be a consequence of the sharp categorization of studies in the service of our meta-analysis, since a more graded perspective is possible, and empirically supported. As mentioned earlier, a recent study documented that cerebellar patients were most impaired when generating the correct sequence of cartoons involving false beliefs, but less so for social routines and non-social events (Van Overwalle *et al.*, 2019a). Likewise, an fMRI study on the same task revealed the highest Crus II activation when stories involved beliefs and less so when they involved social routines and non-social events (Heleven *et al.*, 2019). Thus, to the extent that social actions are automated and require little or no mentalizing, they seem to activate the posterior cerebellum less or not at all.

### Theoretical implications

Some popular explanations of the role of the cerebellum in social cognition did not receive much support in the present study. First, there was little evidence for theories that view the cerebellum as a domain-general modulator of cognitive processes that updates information and sends adaptive feedback to the cerebral cortex (e.g. Bower, 1997; Andreasen and Pierson, 2008). In this view, the cerebellum itself is not responsible for any particular function, but rather facilitates the efficiency by which other neocortical structures perform their own processes. Second, a general time-keeping role of the cerebellum, which coordinates the inputs and outputs from varied sources during processing, as proposed by E *et al.* (2014), is also unlikely. If a timing function plays a role in social thought, it most likely does so during mirroring of observed movements, which was not the focus of this analysis.

In contrast, the present domain-specific results on mentalizing in Crus II are consistent with the view that the general function of the cerebellum is to construct internal models of motor and non-motor sequences of behavior and to directly manipulate through error-feedback the final outputs of sensorimotor, cognitive and emotional functions (Ito and Schuman, 2008; Leggio *et al.*, 2011; Pisotta and Molinari, 2014; Sokolov *et al.*, 2017). This general cerebellar role of sequencing reveals regional differences as a function of the heterogeneous connections to specific functional domains in the cerebral cortex, which contact at precise locations in the cerebellum. As suggested by the present analysis and earlier connectivity studies on the social cerebellum (Van Overwalle *et al.*, 2015b, 2017, 2019c, 2020; Heleven *et al.*, 2019; Van Overwalle and Mariën, 2016), key mentalizing areas in the cerebrum are connected via closed loops with the bilateral Crus II, and so renders this cerebellar area socially relevant. By making plans for sequences in advance and sending corrective feedback about various potential responses of other persons (based on internal cerebellar models), the cerebellum facilitates easy and spontaneous social interaction.

### Clinical implications

The involvement of Crus II in mentalizing offers interesting avenues for clinical diagnosis and treatment of cerebellar and related impairments. First, with respect to diagnosis, this offers tools to predict impairments in specific social mentalizing functions that might be too easily ignored, given the important motoric dysfunctions that are clinically very apparent (D’Mello *et al.*, 2015; D’Mello and Stoodley, 2015; Stoodley *et al.*, 2017). To illustrate, a recent study with autistic adults, analyzing anatomical and structural changes in the cerebellum (Olivito *et al.*, 2018), provided evidence for the mentalizing role of the cerebellum in ASD dysfunctions. The results showed decreased cerebellar grey matter volume in the right Crus II with peak voxel (MNI 29 −73 −43) centered very close to the present ‘sequencing’ ROIs, and this reduced volume was correlated with the degree of autistic traits. Second, this location might become a spot for brain stimulation treatments, using transcranial magnetic stimulation (TMS) or transcranial direct current stimulation (tDCS) (van Dun *et al.*, 2016, 2017). One promising TMS study (Gamond *et al.*, 2017) provided some evidence for reduction of social stereotyping after stimulating the right Crus I (which arguably affected a larger part of the posterior cerebellum). Third, the sequencing hypothesis of social action might offer suggestions for behavioral clinical treatment, because sequencing in social interaction has not attracted a lot of interest in current research on social neuroimaging and treatment.

## Conclusion

The present meta-analysis shows that a domain-specific mentalizing function is supported in the cerebellar Crus II lobule. Within four bilateral ROIs in this area, we found an incidence of 74% of mentalizing functions related to social cognition and self-related emotional cognition. This points to highly specialized areas for mentalizing processes. Importantly, this indicates that the cerebellum has an important social function that has been hereto largely neglected in the scientific community, but one that receives growing evidence from neuroimaging research.

## Supplementary Material

nsaa124_Supp

## Highlights

- This meta-analysis explores the role of the posterior cerebellum in mentalizing.
- Seventy-four percent of studies reflect mentalizing or emotional self-experience in Crus II.
- Results indicate a domain-specific mentalizing functionality in Crus II.

## References in the meta-analysis

AbdulSabur, N. Y., Xu, Y., Liu, S., Chow, H. M., Baxter, M., Carson, J., & Braun, A. R. (2014). Neural correlates and network connectivity underlying narrative production and comprehension: A combined fMRI and PET study. Cortex, 57, 107–127. https://doi.org/10.1016/j.cortex.2014.01.017

Alonso, I., Davachi, L., Valabrègue, R., Lambrecq, V., Dupont, S., & Samson, S. (2016). Neural correlates of binding lyrics and melodies for the encoding of new songs. NeuroImage, 127, 333–345. https://doi.org/10.1016/j.neuroimage.2015.12.018

Amodio, D. M., & Frith, C. D. (2006). Meeting of minds: the medial frontal cortex and social cognition. Nature Reviews. Neuroscience, 7(4), 268–277. https://doi.org/10.1038/nrn1884

Andreasen, N. C., & Pierson, R. (2008). The role of the cerebellum in schizophrenia. Biological Psychiatry, 64(2), 81–88. https://doi.org/10.1016/j.biopsych.2008.01.003

Baetens, K., Ma, N., Steen, J., & Van Overwalle, F. (2013). Involvement of the mentalizing network in social and non-social high construal. Social Cognitive and Affective Neuroscience, in press(6). https://doi.org/10.1093/scan/nst048

Beudel, M., Renken, R., Leenders, K. L., & de Jong, B. M. (2009). Cerebral representations of space and time. NeuroImage, 44(3), 1032–1040. https://doi.org/10.1016/j.neuroimage.2008.09.028

Boiteau, T. W., Bowers, E., Nair, V. A., & Almor, A. (2014). The neural representation of plural discourse entities. Brain and Language, 137, 130–141. https://doi.org/10.1016/j.bandl.2014.08.003

Bonhage, C. E., Fiebach, C. J., Bahlmann, J., & Mueller, J. L. (2014). Brain Signature of Working Memory for Sentence Structure: Enriched Encoding and Facilitated Maintenance. Journal of Cognitive Neuroscience, 26(8), 1654–1671. https://doi.org/10.1162/jocn_a_00566

Bower, J. (1997). Control of sensory data acquisition. International Review of Neurobiology, 41, 489–513.

Brown, B. K., Murrell, J., Karne, H., & Anand, A. (2017). The effects of DAT1 genotype on fMRI activation in an emotional go/no-go task. Brain Imaging and Behavior, 11(1), 185–193. https://doi.org/10.1007/s11682-016-9516-7

Buckner, R., Krienen, F., Castellanos, A., Diaz, J. C., & Yeo, B. T. (2011). The organization of the human cerebellum estimated by intrinsic functional connectivity. Journal of Neurophysiology, 106, 2322–2345. https://doi.org/10.1152/jn.00339.2011

Buhle, J. T., Silvers, J. A., Wager, T. D., Lopez, R., Onyemekwu, C., Kober, H., … Ochsner, K. N. (2013). Cognitive Reappraisal of Emotion: A Meta-Analysis of Human Neuroimaging Studies. Cerebral Cortex, 24(11), 2981–2990. https://doi.org/10.1093/cercor/bht154

Bzdok, D., Langner, R., Hoffstaedter, F., Turetsky, B. I., Zilles, K., & Eickhoff, S. B. (2012). The Modular Neuroarchitecture of Social Judgments on Faces. Cerebral Cortex, 22(4), 951–961. https://doi.org/10.1093/cercor/bhr166

Callan, D., Callan, A., & Jones, J. A. (2014). Speech motor brain regions are differentially recruited during perception of native and foreign-accented phonemes for first and second language listeners. Frontiers in Neuroscience, 8(SEP), 1–15. https://doi.org/10.3389/fnins.2014.00275

Cao, F., Vu, M., Lung Chan, D. H., Lawrence, J. M., Harris, L. N., Guan, Q., … Perfetti, C. A. (2013). Writing affects the brain network of reading in Chinese: A functional magnetic resonance imaging study. Human Brain Mapping, 34(7), 1670–1684. https://doi.org/10.1002/hbm.22017

Case, L. K., Laubacher, C. M., Richards, E. A., Spagnolo, P. A., Olausson, H., & Bushnell, M. C. (2017). Inhibitory rTMS of secondary somatosensory cortex reduces intensity but not pleasantness of gentle touch. Neuroscience Letters, 653, 84–91. https://doi.org/10.1016/j.neulet.2017.05.006

Chaminade, T., Marchant, J. L., Kilner, J., & Frith, C. D. (2012). An fMRI study of joint action-varying levels of cooperation correlates with activity in control networks. Frontiers in Human Neuroscience, 6(June), 179. https://doi.org/10.3389/fnhum.2012.00179

Chen, A. C., Welsh, R. C., Liberzon, I., & Taylor, S. F. (2010). ‘Do I like this person?’ A network analysis of midline cortex during a social preference task. NeuroImage, 51(2), 930–939. https://doi.org/10.1016/j.neuroimage.2010.02.044

Chen, H.-Y., Gilmore, A. W., Nelson, S. M., & McDermott, K. B. (2017). Are There Multiple Kinds of Episodic Memory? An fMRI Investigation Comparing Autobiographical and Recognition Memory Tasks. The Journal of Neuroscience, 37(10), 2764–2775. https://doi.org/10.1523/JNEUROSCI.1534-16.2017

Chow, H. M., Mar, R. A., Xu, Y., Liu, S., Wagage, S., & Braun, A. R. (2014). Embodied Comprehension of Stories: Interactions between Language Regions and Modality-specific Neural Systems Journal of Cognitive Neuroscience, 26(2), 279–295. https://doi.org/10.1162/jocn_a_00487

Collins, D. L., Neelin, P., Peters, T. M., & Evans, A. C. (1994). Automatic 3D intersubject registration of MR volumetric data in standardized Talairach space. Journal of Computer Assisted Tomography, 18, 192–205.

Contreras, J. M., Schirmer, J., Banaji, M. R., & Mitchell, J. P. (2012). Common Brain Regions with Distinct Patterns of Neural Responses during Mentalizing about Groups and Individuals, 1406–1417. https://doi.org/10.1162/jocn

Cramer, S. C., Lastra, L., Lacourse, M. G., & Cohen, M. J. (2005). Brain motor system function after chronic, complete spinal cord injury. Brain, 128(12), 2941–2950. https://doi.org/10.1093/brain/awh648

Crowley, T. J., Dalwani, M. S., Mikulich-Gilbertson, S. K., Du, Y. P., Lejuez, C. W., Raymond, K. M., & Banich, M. T. (2010). Risky Decisions and Their Consequences: Neural Processing by Boys with Antisocial Substance Disorder. PLoS ONE, 5(9), e12835. https://doi.org/10.1371/journal.pone.0012835

Cunningham, W. A., Johnsen, I. R., & Waggoner, A. S. (2011). Orbitofrontal cortex provides cross-modal valuation of self-generated stimuli. Social Cognitive and Affective Neuroscience, 6(3), 286–293. https://doi.org/10.1093/scan/nsq038

Cunningham, W. a., Zelazo, P. D., Packer, D. J., & Van Bavel, J. J. (2007). The Iterative Reprocessing Model: A Multilevel Framework for Attitudes and Evaluation. Social Cognition, 25(5), 736–760. https://doi.org/10.1521/soco.2007.25.5.736

Cunningham, W. a, Raye, C. L., & Johnson, M. K. (2004). Implicit and explicit evaluation: FMRI correlates of valence, emotional intensity, and control in the processing of attitudes. Journal of Cognitive Neuroscience, 16(10), 1717–1729. https://doi.org/10.1162/0898929042947919

D’Argembeau, A., Stawarczyk, D., Majerus, S., Collette, F., Van der Linden, M., Feyers, D., … Salmon, E. (2010). The neural basis of personal goal processing when envisioning future events. Journal of Cognitive Neuroscience, 22(8), 1701–1713. https://doi.org/10.1162/jocn.2009.21314

D’Mello, A. M., Crocetti, D., Mostofsky, S. H., & Stoodley, C. J. (2015). Cerebellar gray matter and lobular volumes correlate with core autism symptoms NeuroImage: Clinical, 7, 631–639. https://doi.org/10.1016/j.nicl.2015.02.007

D’Mello, A. M., & Stoodley, C. J. (2015). Cerebro-cerebellar circuits in autism spectrum disorder. Frontiers in Neuroscience, 9(NOV). https://doi.org/10.3389/fnins.2015.00408

de Diego Balaguer, R., Rodríguez-Fornells, A., Rotte, M., Bahlmann, J., Heinze, H.-J., & Münte, T. F. (2006). Neural circuits subserving the retrieval of stems and grammatical features in regular and irregular verbs. Human Brain Mapping, 27(11), 874–888. https://doi.org/10.1002/hbm.20228

de Manzano, Ö., & Ullén, F. (2012). Goal-independent mechanisms for free response generation: Creative and pseudo-random performance share neural substrates. NeuroImage, 59(1), 772–780. https://doi.org/10.1016/j.neuroimage.2011.07.016

Debbané, M., Badoud, D., Sander, D., Eliez, S., Luyten, P., & Vrtička, P. (2017). Brain activity underlying negative self- and other-perception in adolescents: The role of attachment-derived self-representations. Cognitive, Affective, & Behavioral Neuroscience, 17(3), 554–576. https://doi.org/10.3758/s13415-017-0497-9

Debbané, M., Vrtička, P., Lazouret, M., Badoud, D., Sander, D., & Eliez, S. (2014). Self-reflection and positive schizotypy in the adolescent brain. Schizophrenia Research, 152(1), 65–72. https://doi.org/10.1016/j.schres.2013.06.027

Deming, P., Philippi, C. L., Wolf, R. C., Dargis, M., Kiehl, K. A., & Koenigs, M. (2018). Psychopathic traits linked to alterations in neural activity during personality judgments of self and others. NeuroImage: Clinical, 18(June 2017), 575–581. https://doi.org/10.1016/j.nicl.2018.02.029

Devauchelle, A.-D., Oppenheim, C., Rizzi, L., Dehaene, S., & Pallier, C. (2009). Sentence syntax and content in the human temporal lobe: an fMRI adaptation study in auditory and visual modalities. Journal of Cognitive Neuroscience, 21(5), 1000–1012. https://doi.org/10.1162/jocn.2009.21070

Dodell-Feder, D., DeLisi, L. E., & Hooker, C. I. (2014). Neural disruption to theory of mind predicts daily social functioning in individuals at familial high-risk for schizophrenia. Social Cognitive and Affective Neuroscience, 9(12), 1914–1925. https://doi.org/10.1093/scan/nst186

Doerig, N., Schlumpf, Y., Spinelli, S., Späti, J., Brakowski, J., Quednow, B. B., … Grosse Holtforth, M. (2014). Neural representation and clinically relevant moderators of individualised self-criticism in healthy subjects. Social Cognitive and Affective Neuroscience, 9(9), 1333–1340. https://doi.org/10.1093/scan/nst123

E, K.-H., Chen, S.-H. A., Ho, M.-H. R., & Desmond, J. E. (2014). A meta-analysis of cerebellar contributions to higher cognition from PET and fMRI studies. Human Brain Mapping, 35(2), 593–615. https://doi.org/10.1002/hbm.22194

Eich, E., Nelson, A. L., Leghari, M. A., & Handy, T. C. (2009). Neural systems mediating field and observer memories. Neuropsychologia, 47(11), 2239–2251. https://doi.org/10.1016/j.neuropsychologia.2009.02.019

Eisenberger, N. I., Inagaki, T. K., Rameson, L. T., Mashal, N. M., & Irwin, M. R. (2009). An fMRI study of cytokine-induced depressed mood and social pain: The role of sex differences. NeuroImage, 47(3), 881–890. https://doi.org/10.1016/j.neuroimage.2009.04.040

Engell, A. D., & McCarthy, G. (2013). Probabilistic atlases for face and biological motion perception: An analysis of their reliability and overlap. NeuroImage, 74, 140–151. https://doi.org/10.1016/j.neuroimage.2013.02.025

Engen, H. G., & Singer, T. (2015). Compassion-based emotion regulation up-regulates experienced positive affect and associated neural networks. Social Cognitive and Affective Neuroscience, 10(9), 1291–1301. https://doi.org/10.1093/scan/nsv008

Eriksson, J., Larsson, A., Åhlström, K. R., & Nyberg, L. (2007). Similar Frontal and Distinct Posterior Cortical Regions Mediate Visual and Auditory Perceptual Awareness. Cerebral Cortex, 17(4), 760–765. https://doi.org/10.1093/cercor/bhk029

Evans, K. C., Banzett, R. B., Adams, L., McKay, L., Frackowiak, R. S. J., & Corfield, D. R. (2002). BOLD fMRI Identifies Limbic, Paralimbic, and Cerebellar Activation During Air Hunger. Journal of Neurophysiology, 88(3), 1500–1511. https://doi.org/10.1152/jn.2002.88.3.1500

Fastenrath, M., Coynel, D., Spalek, K., Milnik, A., Gschwind, L., Roozendaal, B., … de Quervain, D. J. F. (2014). Dynamic Modulation of Amygdala-Hippocampal Connectivity by Emotional Arousal. Journal of Neuroscience, 34(42), 13 935–13 947. https://doi.org/10.1523/JNEUROSCI.0786-14.2014

Fedorenko, E., Hsieh, P.-J., Nieto-Castañón, A., Whitfield-Gabrieli, S., & Kanwisher, N. (2010). New Method for fMRI Investigations of Language: Defining ROIs Functionally in Individual Subjects. Journal of Neurophysiology, 104(2), 1177–1194. https://doi.org/10.1152/jn.00032.2010

Fenker, D. B., Schott, B. H., Richardson-Klavehn, A., Heinze, H.-J., & Düzel, E. (2005). Recapitulating emotional context: activity of amygdala, hippocampus and fusiform cortex during recollection and familiarity. European Journal of Neuroscience, 21(7), 1993–1999. https://doi.org/10.1111/j.1460-9568.2005.04033.x

Filippi, M., Riccitelli, G., Meani, A., Falini, A., Comi, G., & Rocca, M. A. (2013). The ‘vegetarian brain’: chatting with monkeys and pigs? Brain Structure and Function, 218(5), 1211–1227. https://doi.org/10.1007/s00429-012-0455-9

Fliessbach, K., Buerger, C., Trautner, P., Elger, C. E., & Weber, B. (2010). Differential effects of semantic processing on memory encoding. Human Brain Mapping, 31(11), NA-NA. https://doi.org/10.1002/hbm.20969

Fogel, S. M., Albouy, G., Vien, C., Popovicci, R., King, B. R., Hoge, R., … Doyon, J. (2014). fMRI and sleep correlates of the age-related impairment in motor memory consolidation. Human Brain Mapping, 35(8), 3625–3645. https://doi.org/10.1002/hbm.22426

Franklin, R. G., & Adams, R. B. (2011). The reward of a good joke: neural correlates of viewing dynamic displays of stand-up comedy. Cognitive, Affective, & Behavioral Neuroscience, 11(4), 508–515. https://doi.org/10.3758/s13415-011-0049-7

Frewen, P. a, Dozois, D. J. a, Neufeld, R. W. J., Densmore, M., Stevens, T. K., & Lanius, R. a. (2011). Neuroimaging social emotional processing in women: fMRI study of script-driven imagery. Social Cognitive and Affective Neuroscience, 6(3), 375–392. https://doi.org/10.1093/scan/nsq047

Fukushima, H., Goto, Y., Maeda, T., Kato, M., & Umeda, S. (2013). Neural Substrates for Judgment of Self-Agency in Ambiguous Situations. PLoS ONE, 8(8), e72267. https://doi.org/10.1371/journal.pone.0072267

Gallese, V., Keysers, C., & Rizzolatti, G. (2004). A unifying view of the basis of social cognition. Trends in Cognitive Sciences, 8(9), 396–403. https://doi.org/10.1016/j.tics.2004.07.002

Gauthier, B., & van Wassenhove, V. (2016). Time Is Not Space: Core Computations and Domain-Specific Networks for Mental Travels. The Journal of Neuroscience, 36(47), 11 891–11 903. https://doi.org/10.1523/JNEUROSCI.1400-16.2016

Gawda, B., & Szepietowska, E. (2016). Trait Anxiety Modulates Brain Activity during Performance of Verbal Fluency Tasks. Frontiers in Behavioral Neuroscience, 10(February), 1–15. https://doi.org/10.3389/fnbeh.2016.00010

Ge, R., Fu, Y., Wang, D., Yao, L., & Long, Z. (2014). Age-related alterations of brain network underlying the retrieval of emotional autobiographical memories: an fMRI study using independent component analysis. Frontiers in Human Neuroscience, 8(August), 1–17. https://doi.org/10.3389/fnhum.2014.00629

Gear, R., Becerra, L., Upadhyay, J., Bishop, J., Wallin, D., Pendse, G., … Borsook, D. (2013). Pain Facilitation Brain Regions Activated by Nalbuphine Are Revealed by Pharmacological fMRI. PLoS ONE, 8(1), e50169. https://doi.org/10.1371/journal.pone.0050169

Grady, C. L., Grigg, O., & Ng, C. (2012). Age differences in default and reward networks during processing of personally relevant information. Neuropsychologia, 50(7), 1682–1697. https://doi.org/10.1016/j.neuropsychologia.2012.03.024

Grahn, J. A., & McAuley, J. D. (2009). Neural bases of individual differences in beat perception. NeuroImage, 47(4), 1894–1903. https://doi.org/10.1016/j.neuroimage.2009.04.039

Green, A. E., Cohen, M. S., Raab, H. A., Yedibalian, C. G., & Gray, J. R. (2015). Frontopolar activity and connectivity support dynamic conscious augmentation of creative state. Human Brain Mapping, 36(3), 923–934. https://doi.org/10.1002/hbm.22676

Groen, W. B., Tesink, C., Petersson, K. M., van Berkum, J., Van Der Gaag, R. J., Hagoort, P., & Buitelaar, J. K. (2010). Semantic, factual, and social language comprehension in adolescents with autism: An FMRI study. Cerebral Cortex, 20(8), 1937–1945. https://doi.org/10.1093/cercor/bhp264

Guell, X., Gabrieli, J. D. E., & Schmahmann, J. D. (2018). Triple representation of language, working memory, social and emotion processing in the cerebellum: convergent evidence from task and seed-based resting-state fMRI analyses in a single large cohort. NeuroImage, 172(January), 437–449. https://doi.org/10.1016/j.neuroimage.2018.01.082

Häberling, I. S., Steinemann, A., & Corballis, M. C. (2016). Cerebral asymmetry for language: Comparing production with comprehension. Neuropsychologia, 80, 17–23. https://doi.org/10.1016/j.neuropsychologia.2015.11.002

Han, S., Mao, L., Qin, J., Friederici, A. D., & Ge, J. (2011). Functional roles and cultural modulations of the medial prefrontal and parietal activity associated with causal attribution. Neuropsychologia, 49(1), 83–91. https://doi.org/10.1016/j.neuropsychologia.2010.11.003

Hartwright, C. E., Apperly, I. A., & Hansen, P. C. (2015). The special case of self-perspective inhibition in mental, but not non-mental, representation. Neuropsychologia, 67, 183–192. https://doi.org/10.1016/j.neuropsychologia.2014.12.015

Hartwright, C. E., Apperly, I. a, & Hansen, P. C. (2012). Multiple roles for executive control in belief-desire reasoning: Distinct neural networks are recruited for self perspective inhibition and complexity of reasoning. NeuroImage, 61(4), 921–930. https://doi.org/10.1016/j.neuroimage.2012.03.012

Heim, S., Grande, M., Meffert, E., Eickhoff, S. B., Schreiber, H., Kukolja, J., … Amunts, K. (2010). Cognitive levels of performance account for hemispheric lateralisation effects in dyslexic and normally reading children. NeuroImage, 53(4), 1346–1358. https://doi.org/10.1016/j.neuroimage.2010.07.009

Hellrung, L., Dietrich, A., Hollmann, M., Pleger, B., Kalberlah, C., Roggenhofer, E., … Horstmann, A. (2018). Intermittent compared to continuous real-time fMRI neurofeedback boosts control over amygdala activation. NeuroImage, 166(October 2017), 198–208. https://doi.org/10.1016/j.neuroimage.2017.10.031

Henckens, M. J. A. G., van Wingen, G. A., Joels, M., & Fernandez, G. (2011). Time-dependent corticosteroid modulation of prefrontal working memory processing. Proceedings of the National Academy of Sciences, 108(14), 5801–5806. https://doi.org/10.1073/pnas.1019128108

Hervais-Adelman, A., Moser-Mercer, B., Michel, C. M., & Golestani, N. (2015). fMRI of Simultaneous Interpretation Reveals the Neural Basis of Extreme Language Control. Cerebral Cortex, 25(12), 4727–4739. https://doi.org/10.1093/cercor/bhu158

Hilty, L., Jäncke, L., Luechinger, R., Boutellier, U., & Lutz, K. (2011). Limitation of physical performance in a muscle fatiguing handgrip exercise is mediated by thalamo-insular activity. Human Brain Mapping, 32(12), 2151–2160. https://doi.org/10.1002/hbm.21177

Hoffman, P., Binney, R. J., & Lambon Ralph, M. A. (2015). Differing contributions of inferior prefrontal and anterior temporal cortex to concrete and abstract conceptual knowledge. Cortex, 63, 250–266. https://doi.org/10.1016/j.cortex.2014.09.001

Holdstock, J. S., Crane, J., Bachorowski, J.-A., & Milner, B. (2010). Equivalent activation of the hippocampus by face-face and face-laugh paired associate learning and recognition. Neuropsychologia, 48(13), 3757–3771. https://doi.org/10.1016/j.neuropsychologia.2010.08.018

Hooker, C. I., Verosky, S. C., Germine, L. T., Knight, R. T., & D’Esposito, M. (2010). Neural activity during social signal perception correlates with self-reported empathy. Brain Research, 1308, 100–113. https://doi.org/10.1016/j.brainres.2009.10.006

Hsu, C.-T., Jacobs, A. M., Citron, F. M. M., & Conrad, M. (2015). The emotion potential of words and passages in reading Harry Potter – An fMRI study. Brain and Language, 142, 96–114. https://doi.org/10.1016/j.bandl.2015.01.011

Iglói, K., Doeller, C. F., Paradis, A.-L., Benchenane, K., Berthoz, A., Burgess, N., & Rondi-Reig, L. (2015). Interaction Between Hippocampus and Cerebellum Crus I in Sequence-Based but not Place-Based Navigation. Cerebral Cortex, 25(11), 4146–4154. https://doi.org/10.1093/cercor/bhu132

Ishizu, T. (2014). A neurobiological enquiry into the origins of our experience of the sublime and beautiful. Frontiers in Human Neuroscience, 8(November), 1–10. https://doi.org/10.3389/fnhum.2014.00891

Ito, H. T., & Schuman, E. M. (2008). Frequency-dependent signal transmission and modulation by neuromodulators. Frontiers in Neuroscience, 2(2), 138–144. https://doi.org/10.3389/neuro.01.027.2008

Jack, A., & Pelphrey, K. A. (2015). Neural Correlates of Animacy Attribution Include Neocerebellum in Healthy Adults. Cerebral Cortex, 25(11), 4240–4247. https://doi.org/10.1093/cercor/bhu146

Jackson, O., & Schacter, D. L. (2004). Encoding activity in anterior medial temporal lobe supports subsequent associative recognition. NeuroImage, 21(1), 456–462. https://doi.org/10.1016/j.neuroimage.2003.09.050

Jackson, R. L., Hoffman, P., Pobric, G., & Lambon Ralph, M. A. (2015). The Nature and Neural Correlates of Semantic Association versus Conceptual Similarity. Cerebral Cortex, 25(11), 4319–4333. https://doi.org/10.1093/cercor/bhv003

Jastorff, J., Huang, Y.-A., Giese, M. A., & Vandenbulcke, M. (2015). Common neural correlates of emotion perception in humans. Human Brain Mapping, 36(10), 4184–4201. https://doi.org/10.1002/hbm.22910

Jiang, D., Edwards, M. G., Mullins, P., & Callow, N. (2015). The neural substrates for the different modalities of movement imagery. Brain and Cognition, 97, 22–31. https://doi.org/10.1016/j.bandc.2015.04.005

Johnston, P., Mayes, A., Hughes, M., & Young, A. W. (2013). Brain networks subserving the evaluation of static and dynamic facial expressions. Cortex, 49(9), 2462–2472. https://doi.org/10.1016/j.cortex.2013.01.002

Jost, K., Khader, P., Burke, M., Bien, S., & Rösler, F. (2009). Dissociating the solution processes of small, large, and zero multiplications by means of fMRI. NeuroImage, 46(1), 308–318. https://doi.org/10.1016/j.neuroimage.2009.01.044

Kandylaki, K. D., Nagels, A., Tune, S., Wiese, R., Bornkessel-Schlesewsky, I., & Kircher, T. (2015). Processing of false belief passages during natural story comprehension: An fMRI study. Human Brain Mapping, 00(February), n/a-n/a. https://doi.org/10.1002/hbm.22907

Kanske, P., Böckler, A., Trautwein, F.-M., & Singer, T. (2015). Dissecting the social brain: Introducing the EmpaToM to reveal distinct neural networks and brain–behavior relations for empathy and Theory of Mind. NeuroImage, 122, 6–19. https://doi.org/10.1016/j.neuroimage.2015.07.082

Kehoe, E. G., Toomey, J. M., Balsters, J. H., & Bokde, A. L. W. (2012). Personality modulates the effects of emotional arousal and valence on brain activation. Social Cognitive and Affective Neuroscience, 7(7), 858–870. https://doi.org/10.1093/scan/nsr059

Keysers, C., & Gazzola, V. (2007). Integrating simulation and theory of mind: From self to social cognition. Trends in Cognitive Sciences, 11, 194–196.

Keysers, Christian, & Perrett, D. I. (2004). Demystifying social cognition: a Hebbian perspective. Trends in Cognitive Sciences, 8(11), 501–507. https://doi.org/10.1016/j.tics.2004.09.005

King, D. R., & Miller, M. B. (2014). Lateral posterior parietal activity during source memory judgments of perceived and imagined events. Neuropsychologia, 53(1), 122–136. https://doi.org/10.1016/j.neuropsychologia.2013.11.006

King, J. a, Hartley, T., Spiers, H. J., Maguire, E. a, & Burgess, N. (2005). Anterior prefrontal involvement in episodic retrieval reflects contextual interference. NeuroImage, 28(1), 256–267. https://doi.org/10.1016/j.neuroimage.2005.05.057

Kleber, B., Veit, R., Birbaumer, N., Gruzelier, J., & Lotze, M. (2010). The Brain of Opera Singers: Experience-Dependent Changes in Functional Activation. Cerebral Cortex, 20(5), 1144–1152. https://doi.org/10.1093/cercor/bhp177

Kompus, K., Hugdahl, K., Öhman, A., Marklund, P., & Nyberg, L. (2009). Distinct control networks for cognition and emotion in the prefrontal cortex. Neuroscience Letters, 467(2), 76–80. https://doi.org/10.1016/j.neulet.2009.10.005

Kong, J., Gollub, R. L., Webb, J. M., Kong, J.-T., Vangel, M. G., & Kwong, K. (2007). Test–retest study of fMRI signal change evoked by electroacupuncture stimulation. NeuroImage, 34(3), 1171–1181. https://doi.org/10.1016/j.neuroimage.2006.10.019

Korn, C. W., Prehn, K., Park, S. Q., Walter, H., & Heekeren, H. R. (2012). Positively Biased Processing of Self-Relevant Social Feedback. Journal of Neuroscience, 32(47), 16 832–16 844. https://doi.org/10.1523/JNEUROSCI.3016-12.2012

Korn, Christoph W., Fan, Y., Zhang, K., Wang, C., Han, S., & Heekeren, H. R. (2014). Cultural influences on social feedback processing of character traits. Frontiers in Human Neuroscience, 8(April), 1–18. https://doi.org/10.3389/fnhum.2014.00192

Krendl, A. C. (2016). An fMRI investigation of the effects of culture on evaluations of stigmatized individuals. NeuroImage, 124, 336–349. https://doi.org/10.1016/j.neuroimage.2015.08.030

Kroemer, N. B., Krebs, L., Kobiella, A., Grimm, O., Vollstädt-Klein, S., Wolfensteller, U., … Smolka, M. N. (2013). (Still) longing for food: Insulin reactivity modulates response to food pictures. Human Brain Mapping, 34(10), 2367–2380. https://doi.org/10.1002/hbm.22071

Laeger, I., Dobel, C., Dannlowski, U., Kugel, H., Grotegerd, D., Kissler, J., … Zwanzger, P. (2012). Amygdala responsiveness to emotional words is modulated by subclinical anxiety and depression. Behavioural Brain Research, 233(2), 508–516. https://doi.org/10.1016/j.bbr.2012.05.036

Lavoie, M.-A., Vistoli, D., Sutliff, S., Jackson, P. L., & Achim, A. M. (2016). Social representations and contextual adjustments as two distinct components of the Theory of Mind brain network: Evidence from the REMICS task. Cortex, 81, 176–191. https://doi.org/10.1016/j.cortex.2016.04.017

Lee, R. J., Coccaro, E. F., Cremers, H., McCarron, R., Lu, S.-F., Brownstein, M. J., & Simon, N. G. (2013). A novel V1a receptor antagonist blocks vasopressin-induced changes in the CNS response to emotional stimuli: an fMRI study. Frontiers in Systems Neuroscience, 7(December), 1–11. https://doi.org/10.3389/fnsys.2013.00100

Lee, T.-W., Dolan, R. J., & Critchley, H. D. (2008). Controlling Emotional Expression: Behavioral and Neural Correlates of Nonimitative Emotional Responses. Cerebral Cortex, 18(1), 104–113. https://doi.org/10.1093/cercor/bhm035

Leggio, M. G., Chiricozzi, F. R., Clausi, S., Tedesco, A. M., & Molinari, M. (2011). The neuropsychological profile of cerebellar damage: The sequencing hypothesis. Cortex, 47(1), 137–144. https://doi.org/10.1016/j.cortex.2009.08.011

Lewis, P. A., Birch, A., Hall, A., & Dunbar, R. I. M. (2017). Higher order intentionality tasks are cognitively more demanding. Social Cognitive and Affective Neuroscience, 12(7), 1063–1071. https://doi.org/10.1093/scan/nsx034

Liljeholm, M., Dunne, S., & O’Doherty, J. P. (2014). Anterior Insula Activity Reflects the Effects of Intentionality on the Anticipation of Aversive Stimulation. Journal of Neuroscience, 34(34), 11 339–11 348. https://doi.org/10.1523/JNEUROSCI.1126-14.2014

Lin, N., Yu, X., Zhao, Y., & Zhang, M. (2016). Functional Anatomy of Recognition of Chinese Multi-Character Words: Convergent Evidence from Effects of Transposable Nonwords, Lexicality, and Word Frequency. PLOS ONE, 11(2), e0149583. https://doi.org/10.1371/journal.pone.0149583

Lindquist, K. a, & Barrett, L. F. (2012). A functional architecture of the human brain: emerging insights from the science of emotion. Trends in Cognitive Sciences, 16(11), 533–540. https://doi.org/10.1016/j.tics.2012.09.005

Lindquist, K. a, Wager, T. D., Kober, H., Bliss-Moreau, E., & Barrett, L. F. (2012). The brain basis of emotion: a meta-analytic review. The Behavioral and Brain Sciences, 35(3), 121–143. https://doi.org/10.1017/S0140525X11000446

Lipp, I., Evans, C. J., Lewis, C., Murphy, K., Wise, R. G., & Caseras, X. (2015). The Relationship between Fearfulness, GABA+, and Fear-Related BOLD Responses in the Insula. PLOS ONE, 10(3), e0120101. https://doi.org/10.1371/journal.pone.0120101

Liu, X., Banich, M. T., Jacobson, B. L., & Tanabe, J. L. (2006). Functional Dissociation of Attentional Selection within PFC: Response and Non-response Related Aspects of Attentional Selection as Ascertained by fMRI. Cerebral Cortex, 16(6), 827–834. https://doi.org/10.1093/cercor/bhj026

Lotze, M., Reimold, M., Heymans, U., Laihinen, A., Patt, M., & Halsband, U. (2009). Reduced Ventrolateral fMRI Response during Observation of Emotional Gestures Related to Reduced Dopamine in the Putamen. Journal of Cognitive Neuroscience, 21(7), 1321–1331.

Maguire, E. A. (2001). The effects of bilateral hippocampal damage on fMRI regional activations and interactions during memory retrieval. Brain, 124(6), 1156–1170. https://doi.org/10.1093/brain/124.6.1156

Maguire, E. a, & Frith, C. D. (2003). Aging affects the engagement of the hippocampus during autobiographical memory retrieval. Brain: A Journal of Neurology, 126(Pt 7), 1511–1523. https://doi.org/10.1093/brain/awg157

Maillet, D., & Rajah, M. N. (2014). Dissociable roles of default-mode regions during episodic encoding. NeuroImage, 89, 244–255. https://doi.org/10.1016/j.neuroimage.2013.11.050

Mainieri, A. G., Peres, J. F. P., Moreira-Almeida, A., Mathiak, K., Habel, U., & Kohn, N. (2017). Neural correlates of psychotic-like experiences during spiritual-trance state. Psychiatry Research: Neuroimaging, 266(August 2016), 101–107. https://doi.org/10.1016/j.pscychresns.2017.06.006

Majerus, S., Salmon, E., & Attout, L. (2013). The Importance of Encoding-Related Neural Dynamics in the Prediction of Inter-Individual Differences in Verbal Working Memory Performance. PLoS ONE, 8(7), e69278. https://doi.org/10.1371/journal.pone.0069278

Makuuchi, M., Kaminaga, T., & Sugishita, M. (2003). Both parietal lobes are involved in drawing: a functional MRI study and implications for constructional apraxia. Cognitive Brain Research, 16(3), 338–347. https://doi.org/10.1016/S0926-6410(02)00302-6

Mar, R. a. (2011). The neural bases of social cognition and story comprehension. Annual Review of Psychology, 62, 103–134. https://doi.org/10.1146/annurev-psych-120709-145406

Martinelli, P., Sperduti, M., & Piolino, P. (2012). Neural substrates of the self-memory system: New insights from a meta-analysis. Human Brain Mapping, 000. https://doi.org/10.1002/hbm.22008

Massat, I., Slama, H., Kavec, M., Linotte, S., Mary, A., Baleriaux, D., … Peigneux, P. (2012). Working Memory-Related Functional Brain Patterns in Never Medicated Children with ADHD. PLoS ONE, 7(11), e49392. https://doi.org/10.1371/journal.pone.0049392

Matsunaga, M., Kawamichi, H., Umemura, T., Hori, R., Shibata, E., Kobayashi, F., … Ohira, H. (2017). Neural and Genetic Correlates of the Social Sharing of Happiness. Frontiers in Neuroscience, 11(DEC), 1–16. https://doi.org/10.3389/fnins.2017.00718

McPherson, B., McMahon, K., Wilson, W., & Copland, D. (2012). ‘I know you can hear me’: Neural correlates of feigned hearing loss. Human Brain Mapping, 33(8), 1964–1972. https://doi.org/10.1002/hbm.21337

Mei, L., Xue, G., Lu, Z.-L., Chen, C., Zhang, M., He, Q., … Dong, Q. (2014). Learning to read words in a new language shapes the neural organization of the prior languages. Neuropsychologia, 65, 156–168. https://doi.org/10.1016/j.neuropsychologia.2014.10.019

Menz, M. M., Buchel, C., & Peters, J. (2012). Sleep Deprivation Is Associated with Attenuated Parametric Valuation and Control Signals in the Midbrain during Value-Based Decision Making. Journal of Neuroscience, 32(20), 6937–6946. https://doi.org/10.1523/JNEUROSCI.3553-11.2012

Meyer, M. L., Spunt, R. P., Berkman, E. T., Taylor, S. E., & Lieberman, M. D. (2012). Evidence for social working memory from a parametric functional MRI study. Proceedings of the National Academy of Sciences of the United States of America, 109(6), 1883–1888. https://doi.org/10.1073/pnas.1121077109

Mitchell, R. L. C. (2007). fMRI delineation of working memory for emotional prosody in the brain: Commonalities with the lexico-semantic emotion network. NeuroImage, 36(3), 1015–1025. https://doi.org/10.1016/j.neuroimage.2007.03.016

Monti, M. M., Coleman, M. R., & Owen, A. M. (2009). Executive functions in the absence of behavior: functional imaging of the minimally conscious state. In Progress in Brain Research (Vol. 177, pp. 249–260). Elsevier. https://doi.org/10.1016/S0079-6123(09)17717-8

Monti, M. M., Osherson, D. N., Martinez, M. J., & Parsons, L. M. (2007). Functional neuroanatomy of deductive inference: a language-independent distributed network. NeuroImage, 37(3), 1005–1016. https://doi.org/10.1016/j.neuroimage.2007.04.069

Moran, J. M., Jolly, E., & Mitchell, J. P. (2012). Social-Cognitive Deficits in Normal Aging. Journal of Neuroscience, 32(16), 5553–5561. https://doi.org/10.1523/JNEUROSCI.5511-11.2012

Morelli, S. a, & Lieberman, M. D. (2013). The role of automaticity and attention in neural processes underlying empathy for happiness, sadness, and anxiety. Frontiers in Human Neuroscience, 7(May), 160. https://doi.org/10.3389/fnhum.2013.00160

Morillon, B., Kell, C. A., & Giraud, A.-L. (2009). Three Stages and Four Neural Systems in Time Estimation. Journal of Neuroscience, 29(47), 14 803–14 811. https://doi.org/10.1523/JNEUROSCI.3222-09.2009

Mutschler, I., Schulze-Bonhage, A., Glauche, V., Demandt, E., Speck, O., & Ball, T. (2007). A Rapid Sound-Action Association Effect in Human Insular Cortex. PLoS ONE, 2(2), e259. https://doi.org/10.1371/journal.pone.0000259

Nagels, A., Kircher, T., Dietsche, B., Backes, H., Marquetand, J., & Krug, A. (2012). Neural processing of overt word generationin healthy individuals: The effect of age and word knowledge. NeuroImage, 61(4), 832–840. https://doi.org/10.1016/j.neuroimage.2012.04.019

Nawa, N. E., & Ando, H. (2014). Classification of Self-Driven Mental Tasks from Whole-Brain Activity Patterns. PLoS ONE, 9(5), e97296. https://doi.org/10.1371/journal.pone.0097296

Okon-Singer, H., Mehnert, J., Hoyer, J., Hellrung, L., Schaare, H. L., Dukart, J., & Villringer, A. (2014). Neural Control of Vascular Reactions: Impact of Emotion and Attention. Journal of Neuroscience, 34(12), 4251–4259. https://doi.org/10.1523/JNEUROSCI.0747-13.2014

Olivito, G., Lupo, M., Laghi, F., Clausi, S., Baiocco, R., Cercignani, M., … Leggio, M. (2018). Lobular patterns of cerebellar resting-state connectivity in adults with Autism Spectrum Disorder. European Journal of Neuroscience, 47(6), 729–735. https://doi.org/10.1111/ejn.13752

Paret, C., Kluetsch, R., Ruf, M., Demirakca, T., Kalisch, R., Schmahl, C., & Ende, G. (2014). Transient and sustained BOLD signal time courses affect the detection of emotion-related brain activation in fMRI. NeuroImage, 103, 522–532. https://doi.org/10.1016/j.neuroimage.2014.08.054

Pauwels, L., Chalavi, S., Gooijers, J., Maes, C., Albouy, G., Sunaert, S., & Swinnen, S. P. (2018). Challenge to Promote Change: The Neural Basis of the Contextual Interference Effect in Young and Older Adults. The Journal of Neuroscience, 38(13), 3333–3345. https://doi.org/10.1523/JNEUROSCI.2640-17.2018

Peake, S. J., Dishion, T. J., Stormshak, E. A., Moore, W. E., & Pfeifer, J. H. (2013). Risk-taking and social exclusion in adolescence: Neural mechanisms underlying peer influences on decision-making. NeuroImage, 82, 23–34. https://doi.org/10.1016/j.neuroimage.2013.05.061

Piazza, M., Pinel, P., Le Bihan, D., & Dehaene, S. (2007). A magnitude code common to numerosities and number symbols in human intraparietal cortex. Neuron, 53(2), 293–305. https://doi.org/10.1016/j.neuron.2006.11.022

Pisotta, I., & Molinari, M. (2014). Cerebellar contribution to feedforward control of locomotion. Frontiers in Human Neuroscience, 8(June), 1–5. https://doi.org/10.3389/fnhum.2014.00475

Poldrack, R. A., Mumford, J. A., Schonberg, T., Kalar, D., Barman, B., & Yarkoni, T. (2012). Discovering Relations Between Mind, Brain, and Mental Disorders Using Topic Mapping. PLoS Computational Biology, 8(10). https://doi.org/10.1371/journal.pcbi.1002707

Potgieser, A. R. E., & de Jong, B. M. (2016). Visuomotor Dissociation in Cerebral Scaling of Size. PLOS ONE, 11(3), e0151484. https://doi.org/10.1371/journal.pone.0151484

Rao, C., Mathur, A., & Singh, N. C. (2013). ‘Cost in Transliteration’: The neurocognitive processing of Romanized writing. Brain and Language, 124(3), 205–212. https://doi.org/10.1016/j.bandl.2012.12.004

Redcay, E., Kleiner, M., & Saxe, R. (2012). Look at this: the neural correlates of initiating and responding to bids for joint attention. Frontiers in Human Neuroscience, 6(June), 1–14. https://doi.org/10.3389/fnhum.2012.00169

Regenbogen, C., Schneider, D. A., Gur, R. E., Schneider, F., Habel, U., & Kellermann, T. (2012). Multimodal human communication — Targeting facial expressions, speech content and prosody. NeuroImage, 60(4), 2346–2356. https://doi.org/10.1016/j.neuroimage.2012.02.043

Rieck, J. R., Rodrigue, K. M., Boylan, M. A., & Kennedy, K. M. (2017). Age-related reduction of BOLD modulation to cognitive difficulty predicts poorer task accuracy and poorer fluid reasoning ability. NeuroImage, 147(December 2016), 262–271. https://doi.org/10.1016/j.neuroimage.2016.12.022

Rosenblau, G., Korn, C. W., & Pelphrey, K. A. (2018). A Computational Account of Optimizing Social Predictions Reveals That Adolescents Are Conservative Learners in Social Contexts. The Journal of Neuroscience, 38(4), 974–988. https://doi.org/10.1523/JNEUROSCI.1044-17.2017

Rueckl, J. G., Paz-Alonso, P. M., Molfese, P. J., Kuo, W.-J., Bick, A., Frost, S. J., … Frost, R. (2015). Universal brain signature of proficient reading: Evidence from four contrasting languages. Proceedings of the National Academy of Sciences, 112(50), 15 510–15 515. https://doi.org/10.1073/pnas.150932-1112

Schaefer, M., Heinze, H.-J., Rotte, M., & Denke, C. (2013). Communicative versus Strategic Rationality: Habermas Theory of Communicative Action and the Social Brain. PLoS ONE, 8(5), e65111. https://doi.org/10.1371/journal.pone.0065111

Schlerf, J., Ivry, R. B., & Diedrichsen, J. (2012). Encoding of Sensory Prediction Errors in the Human Cerebellum. Journal of Neuroscience, 32(14), 4913–4922. https://doi.org/10.1523/JNEUROSCI.4504-11.2012

Schmitgen, M. M., Walter, H., Drost, S., Rückl, S., & Schnell, K. (2016). Stimulus-dependent amygdala involvement in affective theory of mind generation. NeuroImage, 129, 450–459. https://doi.org/10.1016/j.neuroimage.2016.01.029

Schneider, K., Pauly, K. D., Gossen, A., Mevissen, L., Michel, T. M., Gur, R. C., … Habel, U. (2013). Neural correlates of moral reasoning in autism spectrum disorder. Social Cognitive and Affective Neuroscience, 8(6), 702–710. https://doi.org/10.1093/scan/nss051

Schneider, M., Debbané, M., Lagioia, A., Salomon, R., D’Argembeau, A., & Eliez, S. (2012). Comparing the neural bases of self-referential processing in typically developing and 22q11.2 adolescents. Developmental Cognitive Neuroscience, 2(2), 277–289. https://doi.org/10.1016/j.dcn.2011.12.004

Schnell, K., Bluschke, S., Konradt, B., & Walter, H. (2011). Functional relations of empathy and mentalizing: an fMRI study on the neural basis of cognitive empathy. NeuroImage, 54(2), 1743–1754. https://doi.org/10.1016/j.neuroimage.2010.08.024

Schurz, M., Radua, J., Aichhorn, M., Richlan, F., & Perner, J. (2014). Fractionating theory of mind: A meta-analysis of functional brain imaging studies. Neuroscience and Biobehavioral Reviews, 42, 9–34. https://doi.org/10.1016/j.neubiorev.2014.01.009

Seghier, M. L., Josse, G., Leff, A. P., & Price, C. J. (2011). Lateralization is Predicted by Reduced Coupling from the Left to Right Prefrontal Cortex during Semantic Decisions on Written Words. Cerebral Cortex, 21(7), 1519–1531. https://doi.org/10.1093/cercor/bhq203

Servaas, M. N., Aleman, A., Marsman, J.-B. C., Renken, R. J., Riese, H., & Ormel, J. (2015). Lower dorsal striatum activation in association with neuroticism during the acceptance of unfair offers. Cognitive, Affective, & Behavioral Neuroscience, 15(3), 537–552. https://doi.org/10.3758/s13415-015-0342-y

Shenhav, A., & Greene, J. D. (2014). Integrative Moral Judgment: Dissociating the Roles of the Amygdala and Ventromedial Prefrontal Cortex. Journal of Neuroscience, 34(13), 4741–4749. https://doi.org/10.1523/JNEUROSCI.3390-13.2014

Sokolov, A. A., Miall, R. C., & Ivry, R. B. (2017). The Cerebellum: Adaptive Prediction for Movement and Cognition. Trends in Cognitive Sciences, xx, 1–20. https://doi.org/10.1016/j.tics.2017.02.005

Spreng, R. N., DuPre, E., Selarka, D., Garcia, J., Gojkovic, S., Mildner, J., … Turner, G. R. (2014). Goal-Congruent Default Network Activity Facilitates Cognitive Control. Journal of Neuroscience, 34(42), 14 108–14 114. https://doi.org/10.1523/JNEUROSCI.2815-14.2014

Spreng, R Nathan, Mar, R. a, & Kim, A. S. N. (2008). The Common Neural Basis of Autobiographical Memory, Prospection, Navigation, Theory of Mind, and the Default Mode: A Quantitative Meta-analysis. Journal of Cognitive Neuroscience, 21(3), 489–510. https://doi.org/10.1162/jocn.2008.21029

Spunt, R. P., & Adolphs, R. (2014). Validating the Why/How contrast for functional MRI studies of Theory of Mind. NeuroImage, 99(MAY 2014), 301–311. https://doi.org/10.1016/j.neuroimage.2014.05.023

Spunt, R. P., Falk, E. B., & Lieberman, M. D. (2010). Dissociable neural systems support retrieval of how and why action knowledge. Psychological Science, 21(11), 1593–1598. https://doi.org/10.1177/0956797610386618

Spunt, R. P., & Lieberman, M. D. (2012). Dissociating modality-specific and supramodal neural systems for action understanding. Journal of Neuroscience, 32(10), 3575–3583. https://doi.org/10.1523/JNEUROSCI.5715-11.2012

Spunt, R. P., Satpute, A. B., & Lieberman, M. D. (2011). Identifying the what, why, and how of an observed action: an fMRI study of mentalizing and mechanizing during action observation. Journal of Cognitive Neuroscience, 23(1), 63–74. https://doi.org/10.1162/jocn.2010.21446

St. Jacques, P. L., Carpenter, A. C., Szpunar, K. K., & Schacter, D. L. (2018). Remembering and imagining alternative versions of the personal past. Neuropsychologia, 110(June 2017), 170–179. https://doi.org/10.1016/j.neuropsychologia.2017.06.015

Stevens, M. C., Calhoun, V. D., & Kiehl, K. A. (2005). Hemispheric differences in hemodynamics elicited by auditory oddball stimuli. NeuroImage, 26(3), 782–792. https://doi.org/10.1016/j.neuroimage.2005.02.044

Stoodley, C. J., D’Mello, A. M., Ellegood, J., Jakkamsetti, V., Liu, P., Nebel, M. B., … Tsai, P. T. (2017). Altered cerebellar connectivity in autism and cerebellar-mediated rescue of autism-related behaviors in mice. Nature Neuroscience. https://doi.org/10.1038/s41593-017-0004-1

Stoodley, C. J., & Schmahmann, J. D. (2009). Functional topography in the human cerebellum: a meta-analysis of neuroimaging studies. NeuroImage, 44(2), 489–501. https://doi.org/10.1016/j.neuroimage.2008.08.039

Strandberg, M., Elfgren, C., Mannfolk, P., Olsrud, J., Stenberg, L., van Westen, D., … Källén, K. (2011). fMRI memory assessment in healthy subjects: a new approach to view lateralization data at an individual level. Brain Imaging and Behavior, 5(1), 1–11. https://doi.org/10.1007/s11682-010-9106-z

Summerfield, J. J., Hassabis, D., & Maguire, E. a. (2009). Cortical midline involvement in autobiographical memory. NeuroImage, 44(3), 1188–1200. https://doi.org/10.1016/j.neuroimage.2008.09.033

Svoboda, E., McKinnon, M., & Levine, B. (2006). The functional neuroanatomy of autobiographical memory: a meta-analysis. Neuropsychologia, 44(12), 2189–2208. Retrieved from http://www.sciencedirect.com/science/article/pii/S0028393206-002090

Talairach, J., & Tournoux, P. (1988). Co-planar stereotaxic atlas of the human brain. Stuttgart: Thieme.

Tesink, C. M. J. Y., Buitelaar, J. K., Petersson, K. M., van der Gaag, R. J., Teunisse, J.-P., & Hagoort, P. (2011). Neural correlates of language comprehension in autism spectrum disorders: When language conflicts with world knowledge. Neuropsychologia, 49(5), 1095–1104. https://doi.org/10.1016/j.neuropsychologia.2011.01.018

Thürling, M., Hautzel, H., Küper, M., Stefanescu, M. R., Maderwald, S., Ladd, M. E., & Timmann, D. (2012). Involvement of the cerebellar cortex and nuclei in verbal and visuospatial working memory: A 7T fMRI study. NeuroImage, 62(3), 1537–1550. https://doi.org/10.1016/j.neuroimage.2012.05.037

Thürling, M., Küper, M., Stefanescu, R., Maderwald, S., Gizewski, E. R., Ladd, M. E., & Timmann, D. (2011). Activation of the dentate nucleus in a verb generation task: A 7T MRI study. NeuroImage, 57(3), 1184–1191. https://doi.org/10.1016/j.neuroimage.2011.05.045

Tzvi, E., Zimmermann, C., Bey, R., Münte, T. F., Nitschke, M., & Krämer, U. M. (2017). Cerebellar degeneration affects cortico-cortical connectivity in motor learning networks. NeuroImage: Clinical, 16(March), 66–78. https://doi.org/10.1016/j.nicl.2017.07.012

Uchiyama, H., Seki, A., Kageyama, H., Saito, D. N., Koeda, T., Ohno, K., & Sadato, N. (2006). Neural substrates of sarcasm: A functional magnetic-resonance imaging study. Brain Research, 1124(1), 100–110. https://doi.org/10.1016/j.brainres.2006.09.088

Uddin, L. Q., Iacoboni, M., Lange, C., & Keenan, J. P. (2007). The self and social cognition: the role of cortical midline structures and mirror neurons. Trends in Cognitive Sciences, 11(4), 153–157. https://doi.org/10.1016/j.tics.2007.01.001

van Buuren, M., Kroes, M. C. W., Wagner, I. C., Genzel, L., Morris, R. G. M., & Fernandez, G. (2014). Initial Investigation of the Effects of an Experimentally Learned Schema on Spatial Associative Memory in Humans. Journal of Neuroscience, 34(50), 16 662–16 670. https://doi.org/10.1523/JNEUROSCI.2365-14.2014

van der Laan, L. N., de Ridder, D. T. D., Viergever, M. A., & Smeets, P. A. M. (2014). Activation in inhibitory brain regions during food choice correlates with temptation strength and self-regulatory success in weight-concerned women. Frontiers in Neuroscience, 8(SEP), 1–10. https://doi.org/10.3389/fnins.2014.00308

van Dun, K., Bodranghien, F. C. A. A., Mariën, P., & Manto, M. U. (2016). tDCS of the Cerebellum: Where Do We Stand in 2016? Technical Issues and Critical Review of the Literature. Frontiers in Human Neuroscience, 10(May). https://doi.org/10.3389/fnhum.2016.00199

van Dun, K., Bodranghien, F., Manto, M., & Mariën, P. (2017). Targeting the cerebellum by noninvasive neurostimulation: A review. Submitted, 16(3), 695–741. https://doi.org/10.1007/s12311-016-0840-7

Van Hoeck, N., Begtas, E., Steen, J., Kestemont, J., Vandekerckhove, M., & Van Overwalle, F. (2013). False belief and counterfactual reasoning in a social environment. NeuroImage, 90C, 315–325. https://doi.org/10.1016/j.neuroimage.2013.12.043

Van Hoeck, N., Ma, N., Ampe, L., Baetens, K., Vandekerckhove, M., & Van Overwalle, F. (2013). Counterfactual thinking: an fMRI study on changing the past for a better future. Social Cognitive and Affective Neuroscience, 8(5), 556–564. https://doi.org/10.1093/scan/nss031

Van Overwalle, F. (2009). Social cognition and the brain: a meta-analysis. Human Brain Mapping, 30(3), 829–858. https://doi.org/10.1002/hbm.20547

Van Overwalle, F. (2011). A dissociation between social mentalizing and general reasoning. NeuroImage, 54(2), 1589–1599. https://doi.org/10.1016/j.neuroimage.2010.09.043

Van Overwalle, F., & Baetens, K. (2009). Under-standing others’ actions and goals by mirror and mentalizing systems: a meta-analysis. NeuroImage, 48(3), 564–584. https://doi.org/10.1016/j.neuroimage.2009.06.009

Van Overwalle, F., Baetens, K., Mariën, P., & Vandekerckhove, M. (2014). Social cognition and the cerebellum: A meta-analysis of over 350 fMRI studies. NeuroImage, 86, 554–572. https://doi.org/10.1016/j.neuroimage.2013.09.033

Van Overwalle, F., Baetens, K., Mariën, P., & Vandekerckhove, M. (2015). Cerebellar areas dedicated to social cognition? A comparison of meta-analytic and connectivity results. Social Neuroscience, 10(4), 337–344. https://doi.org/10.1080/17470919.2015.1005666

Van Overwalle, F., D’aes, T., & Mariën, P. (2015). Social cognition and the cerebellum: A meta-analytic connectivity analysis. Human Brain Mapping, 36(12), 5137–5154. https://doi.org/10.1002/hbm.23002

Van Overwalle, F., De Coninck, S., Heleven, E., Perrotta, G., Taib, N. O. Ben, Manto, M., & Mariën, P. (2019). The role of the Cerebellum in reconstructing Social Action Sequences: A Pilot Study. Social Cognitive and Affective Neuroscience. https://doi.org/10.1093/scan/nsz032

Van Overwalle, F., Heleven, E., Ma, N., & Mariën, P. (2017). Tell me twice: A Multi-Study analysis of the functional connectivity between the cerebrum and cerebellum after repeated trait information. NeuroImage, 144(A), 241–252. https://doi.org/10.1016/j.neuroimage.2016.08.046

Van Overwalle, F., Manto, M., Leggio, M., & Delgado-García, J. M. (2019). The sequencing process generated by the cerebellum crucially contributes to social interactions. Medical Hypotheses. https://doi.org/10.1016/j.mehy.2019.05.014

Van Overwalle, F., & Mariën, P. (2016). Functional connectivity between the cerebrum and cerebellum in social cognition: A multi-study analysis. NeuroImage, 124, 248–255. https://doi.org/10.1016/j.neuroimage.2015.09.001

Van Overwalle, F., Van de Steen, F., & Mariën, P. (2019). Dynamic causal modeling of the effective connectivity between the cerebrum and cerebellum in social mentalizing across five studies. Cognitive, Affective, & Behavioral Neuroscience, 19(1), 211–223. https://doi.org/10.3758/s13415-018-00659-y

Van Overwalle, F., Van de Steen, F., van Dun, K., & Heleven, E. (2019). Connectivity between the Cerebrum and Cerebellum during Social and Non-Social Sequencing using Dynamic Causal Modelling. Unpublished Manuscript.

Vanderhasselt, M.-A., Baeken, C., Van Schuerbeek, P., Luypaert, R., De Mey, J., & De Raedt, R. (2013). How brooding minds inhibit negative material: An event-related fMRI study. Brain and Cognition, 81(3), 352–359. https://doi.org/10.1016/j.bandc.2013.01.007

Vanderhasselt, M.-A., Baeken, C., Van Schuerbeek, P., Luypaert, R., & De Raedt, R. (2013). Inter-individual differences in the habitual use of cognitive reappraisal and expressive suppression are associated with variations in prefrontal cognitive control for emotional information: An event related fMRI study. Biological Psychology, 92(3), 433–439. https://doi.org/10.1016/j.biopsycho.2012.03.005

Vetter, N. C., Weigelt, S., Döhnel, K., Smolka, M. N., & Kliegel, M. (2014). Ongoing neural development of affective theory of mind in adolescence. Social Cognitive and Affective Neuroscience, 9(7), 1022–1029. https://doi.org/10.1093/scan/nst081

Viard, A., Doeller, C. F., Hartley, T., Bird, C. M., & Burgess, N. (2011). Anterior Hippocampus and Goal-Directed Spatial Decision Making. Journal of Neuroscience, 31(12), 4613–4621. https://doi.org/10.1523/JNEUROSCI.4640-10.2011

Vilberg, K. L., & Rugg, M. D. (2009). An investigation of the effects of relative probability of old and new test items on the neural correlates of successful and unsuccessful source memory. NeuroImage, 45(2), 562–571. https://doi.org/10.1016/j.neuroimage.2008.12.020

Völlm, B., Richardson, P., McKie, S., Elliott, R., Dolan, M., & Deakin, B. (2007). Neuronal correlates of reward and loss in Cluster B personality disorders: A functional magnetic resonance imaging study. Psychiatry Research: Neuroimaging, 156(2), 151–167. https://doi.org/10.1016/j.pscychresns.2007.04.008

Vorobyev, V., Kwon, M. S., Moe, D., Parkkola, R., & Hämäläinen, H. (2015). Risk-Taking Behavior in a Computerized Driving Task: Brain Activation Correlates of Decision-Making, Outcome, and Peer Influence in Male Adolescents. PLOS ONE, 10(6), e0129516. https://doi.org/10.1371/journal.pone.0129516

Wagner, I. C., van Buuren, M., Bovy, L., & Fernández, G. (2016). Parallel Engagement of Regions Associated with Encoding and Later Retrieval Forms Durable Memories. The Jour- nal of Neuroscience, 36(30), 7985–7995. https://doi.org/10.1523/JNEUROSCI.0830-16.2016

Wallmark, Z., Deblieck, C., & Iacoboni, M. (2018). Neurophysiological Effects of Trait Empathy in Music Listening. Frontiers in Behavioral Neuroscience, 12(April), 1–19. https://doi.org/10.3389/fnbeh.2018.00066

Wang, Q., Luo, S., Monterosso, J., Zhang, J., Fang, X., Dong, Q., & Xue, G. (2014). Distributed Value Representation in the Medial Prefrontal Cortex during Intertemporal Choices. Journal of Neuroscience, 34(22), 7522–7530. https://doi.org/10.1523/JNEUROSCI.0351-14.2014

Wang, T. H., Johnson, J. D., de Chastelaine, M., Donley, B. E., & Rugg, M. D. (2016). The Effects of Age on the Neural Correlates of Recollection Success, Recollection-Related Cortical Reinstatement, and Post-Retrieval Monitoring. Cerebral Cortex, 26(4), 1698–1714. https://doi.org/10.1093/cercor/bhu333

Wang, T., Mo, L., Mo, C., Tan, L. H., Cant, J. S., Zhong, L., & Cupchik, G. (2015). Is moral beauty different from facial beauty? Evidence from an fMRI study. Social Cognitive and Affective Neuroscience, 10(6), 814–823. https://doi.org/10.1093/scan/nsu123

Weidner, R., Krummenacher, J., Reimann, B., Müller, H. J., & Fink, G. R. (2009). Sources of top-down control in visual search. Journal of Cognitive Neuroscience, 21(11), 2100–2113. https://doi.org/10.1162/jocn.2008.21173

Weinstein, S., Werker, J. F., Vouloumanos, A., Woodward, T. S., & Ngan, E. T. C. (2006). Do you hear what I hear? Neural correlates of thought disorder during listening to speech in schizophrenia. Schizophrenia Research, 86(1–3), 130–137. https://doi.org/10.1016/j.schres.2006.05.011

Willems, R. M., Clevis, K., & Hagoort, P. (2010). Add a picture for suspense: neural correlates of the interaction between language and visual information in the perception of fear. Social Cognitive and Affective Neuroscience. https://doi.org/10.1093/scan/nsq050

Wilson, S. M., Molnar-Szakacs, I., & Iacoboni, M. (2008). Beyond superior temporal cortex: intersubject correlations in narrative speech comprehension. Cerebral Cortex (New York, N.Y. : 1991), 18(1), 230–242. https://doi.org/10.1093/cercor/bhm049

Xu, J., Kemeny, S., Park, G., Frattali, C., & Braun, A. (2005). Language in context: emergent features of word, sentence, and narrative comprehension. NeuroImage, 25(3), 1002–1015. https://doi.org/10.1016/j.neuroimage.2004.12.013

Xu, P., Gu, R., Broster, L. S., Wu, R., Van Dam, N. T., Jiang, Y., … Luo, Y. -j. (2013). Neural Basis of Emotional Decision Making in Trait Anxiety. Journal of Neuroscience, 33(47), 18 641–18 653. https://doi.org/10.1523/JNEUROSCI.1253-13.2013

Xu, R., Yang, J., Feng, C., Wu, H., Huang, R., Yang, Q., … Luo, Y. (2018). Time is nothing: emotional consistency of autobiographical memory and its neural basis. Brain Imaging and Behavior, 12(4), 1053–1066. https://doi.org/10.1007/s11682-017-9778-8

Yeo, B. T. T., Krienen, F. M., Sepulcre, J., Sabuncu, M. R., Lashkari, D., Hollinshead, M., … Buckner, R. L. (2011). The organization of the human cerebral cortex estimated by intrinsic functional connectivity. Journal of Neurophysiology, 106(3), 1125–1165. https://doi.org/10.1152/jn.00338.2011

Zaki, J., Davis, J. I., & Ochsner, K. N. (2012). Overlapping activity in anterior insula during interoception and emotional experience. NeuroImage, 62(1), 493–499. https://doi.org/10.1016/j.neuroimage.2012.05.012
